# Spatial transcriptomics reveals an unexpected impact of tau and tau pathology on the expression of transthyretin

**DOI:** 10.3389/fnagi.2025.1656850

**Published:** 2025-10-31

**Authors:** Deborah L. Croteau, Jose Fernandez Navarro, Thomas Comptdaer, Zaneta Andrusivova, Aleksandra Jurek, Eliette Bonnefoy, Luc Buée, Vilhelm A. Bohr, Joakim Lundeberg, Marie-Christine Galas

**Affiliations:** ^1^National Institute on Aging, Section on DNA Repair, Baltimore, MD, United States; ^2^National Institute on Aging, Laboratory of Genomics and Genetics, Computational Biology and Genomic Core, Baltimore, MD, United States; ^3^Science for Life Laboratory, Division of Gene Technology, KTH Royal Institute of Technology, Stockholm, Sweden; ^4^Univ. Lille, Inserm, CHU Lille, CNRS, LilNCog-Lille Neuroscience & Cognition, Lille, France; ^5^Université Paris Cité, CNRS, Inserm, Institut Cochin, Paris, France; ^6^Department of ICMM, University of Copenhagen, Copenhagen, Denmark

**Keywords:** spatial transcriptomics, tau, tauopathies, transthyretin, amyloid beta, aggregation

## Abstract

**Introduction:**

RNA expression is modulated by tau. We used two mouse models, THY-Tau22 mice, which express pro-aggregation tau, and TauKO mice, which are null for tau, to improve our understanding of tau-altered mRNA expression in brain.

**Methods:**

Spatial transcriptomics on Tau22 and TauKO mice were used to interrogate regional mRNA expression changes. We focused on mRNA expression changes in the hippocampus and ventricles; two regions altered early in Alzheimer’s disease.

**Results:**

We identified the transthyretin mRNA, *Ttr*, as being dysregulated in a tau-dependent manner. Immunofluorescence (IF) revealed increased TTR protein expression in THY-Tau22 mice and lowered expression in TauKO mice in the choroid plexus epithelial cells.

**Conclusion:**

As TTR is involved in the clearance of Aβ and the prevention of Aβ aggregation, we evaluated endogenous mouse Aβ in TauKO mice and observed increased Aβ deposits. Our study reveals a hitherto unknown regulatory role of tau on *Ttr* mRNA and protein expression, which may participate in a feedback loop contributing to Aβ disease progression.

## 1 Introduction

One hallmark of many neurodegenerative diseases known as tauopathies, including Alzheimer’s disease (AD), is neurofibrillary tangles composed of hyperphosphorylated tau, which correlate with dementia and synaptic and neuronal loss (DeTure and Dickson, 2019). Although tau was first described as a microtubule stabilizer, it is a highly multifunctional protein (Sotiropoulos et al., 2017; Tracy et al., 2022). Deciphering the complete set of physiological roles of tau and its dysregulation is critical to refining our understanding of the etiology of these pathologies.

RNA expression changes contribute to Tau-related neurodegenerative diseases (Chung et al., 2021). Among the growing number of features attributed to tau, is that it can modulate chromatin structure, nuclear tension, and the expression of various RNAs, and that tau pathology can trigger RNA expression alterations (Ali et al., 2024; Benhelli-Mokrani et al., 2018; Maina et al., 2018; Mansuroglu et al., 2016; Sohn et al., 2023; Wes et al., 2014; Woo et al., 2010). However, until now only global transcriptomics approaches have been used to investigate the ability of tau or tau pathology to act on RNA expression, but these techniques lack information concerning spatial tissue-level regulation. Spatial transcriptomics (ST) is well-suited to analyze RNA expression changes in a spatially resolved unbiased manner. ST has been successfully used in the context of AD-like mouse models and human brains (Chen et al., 2022; Chen et al., 2020; Choi et al., 2023; Das et al., 2024; Mathys et al., 2019; Navarro et al., 2020; Yu et al., 2024; Zou et al., 2024) but so far it has not been applied to analyze the influence of tau, and its pathological forms, on RNA expression in a spatial context.

Neurodegeneration is complex and mostly brain-region specific. Fortunately, ST analyses have the capacity to resolve spatially differentially expressed RNAs in distinct brain regions. In AD, there is an ordered progression of AD biomarkers specific to the brain and cerebral spinal fluid (CSF) (Jack et al., 2024). Two close brain regions impaired early in AD are the hippocampus and adjacent brain ventricles (Coupe et al., 2019). The hippocampus plays a central role in cognition, while the ventricles, where CSF is produced, lie at the interface between the peripheral circulation and the central nervous system. Ventricles enclose the choroid plexus (CP), which is composed of a monolayer of epithelial cells surrounding a highly vascularized connective tissue layer with permeable capillaries. CP epithelial cells form the blood–cerebrospinal fluid barrier, which strictly regulates the exchange of factors between the blood and CSF. Tau protein is physiologically expressed in the hippocampus but has not been detected in the CP (Uhlen et al., 2015). However, in AD brains, tau pathology affects both the hippocampus and the CP (Chung et al., 2021; Raha-Chowdhury et al., 2019). Further, defects in the CP have recently been defined as a new subgroup of AD based on mass spectrometry of CSF proteins comparing AD patients and controls (Tijms et al., 2024).

One of the proteins that is a marker for the CP is transthyretin (TTR). The multifunctional TTR protein is primarily synthesized in CP epithelial cells lining the lumen of the lateral ventricles (Dickson et al., 1986; Stauder et al., 1986). TTR is also expressed in the liver and retinal epithelium. It is a secreted protein and is secreted from the liver into the serum and from CP cells into the CSF. TTR is a major thyroid hormone carrier and in association with retinol-binding protein transports retinols [for recent review see Gertz et al. (2025)]. TTR has neuroprotective functions under some conditions, as it is important for neurite outgrowth and neurogenesis (Gomes et al., 2016). TTR plays a role in memory as evidenced from TTR-null mice (Brouillette and Quirion, 2008). Additionally, mutations in TTR contribute to the genetic disorder transthyretin amyloidosis in humans (Gertz et al., 2025). TTR is also an extracellular chaperone with neuroprotective roles in stress conditions (Buxbaum and Johansson, 2017; Gomes et al., 2016; Liz et al., 2020; Santos et al., 2010). Notably, TTR has been linked to AD, as it binds to amyloid beta peptide (Abeta, Aβ), sequesters it, and prevents its aggregation (Buxbaum, 2023; Buxbaum et al., 2008; Cascella et al., 2013; Ciccone et al., 2020; Costa et al., 2008; Gonzalez-Marrero et al., 2015; Iqbal, 2018; Li et al., 2013; Liz et al., 2020; Nilsson et al., 2018; Schwarzman et al., 1994; Ueda, 2022; West et al., 2021).

The goal of this study was to use ST to identify RNAs that were differentially expressed (DE) between regions in brain sections from transgenic mouse models of tau pathology THY-Tau22 (Tau22) and tau deletion (TauKO). Here, we have used 12 months-old Tau22, at the peak of tau pathology, TauKO, and their respective WT littermate mice, and applied ST to identify tau-dependent RNA expression changes in the aged brain.

ST analysis revealed the dysregulated expression of *Ttr* RNA in Tau22 mice. Using IF, we observed TTR downregulation in CP epithelial cells from TauKO mouse brains indicating that tau may positively regulate TTR expression. This finding was correlated with increased Aβ deposits in the hippocampus, suggesting that tau- regulated TTR participates to prevent Aβ accumulation in the brain. The absence of Aβ accumulation in aged Tau22 mouse brains correlates with increased TTR in CP epithelial cells. Altogether this study highlights *i-* a modulatory effect of tau and tau pathology on RNA expression, including the RNA encoding TTR, and *ii-* an unexpected inhibitory role of tau on endogenous Aβ peptide accumulation in mouse brains. Combined ST and IF reveal a hitherto unknown regulatory role of tau on *Ttr* RNA and protein expression, which may contribute to a feedback loop on Aβ disease progression.

## 2 Materials and methods

### 2.1 Mice

This study employed all female mice. The THY-Tau22 transgenic mouse model was generated to model AD-like tau pathology that is associated with learning and memory deficits (Schindowski et al., 2006). Tau22 mice overexpress the 4R *tau* RNA mutated at G272V and P301S and develop, mainly in the hippocampus, aggregation of hyperphosphorylated tau in addition to progressive tau-related neuropathology. In Tau22 mice, hyperphosphorylated and aggregated forms of tau first appear in the CA1 subfield and are present throughout the hippocampus at the peak of pathology at 12 months (Schindowski et al., 2006). Tau22 mice display learning and memory deficits and, although long-term potentiation (LTP) is intact, they show changes in NMDA-dependent long-term depression (LTD), and hippocampal synaptic plasticity (Schindowski et al., 2006; Van der Jeugd et al., 2011).

TauKO mice are meant to mimic the loss of tau protein from its normal physiological compartments (Tucker et al., 2001). The behavioral findings in the TauKO model include loss of contextual and cued fear conditioning, but normal motor, exploratory, and anxiety behaviors (Ahmed et al., 2014). Our TauKO mice display better spatial learning than controls in the water maze test. Additionally, in electrophysiological tests TauKO mice showed no change in basal synaptic transmission and paired-pulse facilitation from the CA1 hippocampal region. However, LTP, but not LTD, showed severe deficits.

All animals were kept in standard animal cages (12 h/12 h light/dark cycle, at 22 °C), with ad libitum access to food and water. The animals were maintained in compliance with institutional protocols (Comité d’éthique en expérimentation animale du Nord Pas-de-Calais, no. 0508003). All the animal experiments were performed in compliance with and following the approval of the local Animal Ethical Committee (agreement #12787–2, 015,101,320,441,671 v9 from CEEA75, Lille, France), standards for the care and use of laboratory animals, and the French and European Community rules.

### 2.2 Ethics approval

Animals were maintained in compliance with institutional protocols (Comité d’éthique en expérimentation animale du Nord Pas-de-Calais, no. 0508003). All the animal experiments were performed in compliance with and following the approval of the local Animal Ethical Committee (agreement #12787–2, 015, 101, 320, 441, 671 v9nfrom CEEA75, Lille, France), standards for the care and use of laboratory animals, and the French and European Community rules.

### 2.3 Tissue collection and sectioning

Adult mice were sacrificed, and the brains were removed from the cranial cavity, embedded in OCT, and snap-frozen in isopentane pre-cooled with dry ice and liquid nitrogen. The left hemispheres were sectioned on the cryostat at 10 μm thickness. Sections were placed on the spatially barcoded arrays with one section per well.

### 2.4 Fixation, staining, and imaging

Sections were fixed in 3.6–3.8% formaldehyde (Sigma) in PBS, washed in PBS, then treated for 1 min with isopropanol and air-dried. To stain the tissue, sections were incubated in Mayer’s Hematoxylin (Dako) for 7 min, then Bluing buffer for 2 min and Eosin (Sigma) for 20 s. After drying, the slides were mounted with 85% glycerol and images of sections were taken using Metafer Slide Scanning Platform (Metasystems). Raw images were stitched together using VSlide software (Metasystems).

### 2.5 Tissue pre- and permeabilization

To pre-permeabilize the tissue, sections were incubated for 20 min at 37 °C with 0.5 U/ul collagenase (Thermofisher) in HBSS buffer mixed with 0.2 ug/ul BSA (NEB). Following washing in 0.1x SSC buffer (Sigma), sections were permeabilized with 0.1% pepsin/HCl (Sigma) at 37 °C for 10 and 6 min, respectively. Then, the sections were washed with 0.1x SSC buffer.

### 2.6 Reverse transcription and library preparation

After permeabilization, reverse transcription mix containing Superscript III reverse transcriptase (Thermofisher) was added to each section and incubated overnight at 42 °C as described previously (Stahl et al., 2016). Next, to remove tissue from the slide, sections were incubated for 1 h at 56 °C with Proteinase K in PKD buffer (both from Qiagen). Surface probes with bound mRNA/cDNA were then cleaved from the slide by USER enzyme (NEB) (Stahl et al., 2016). Released probes were collected from each well and transferred to separate tubes. Next, 2nd strand synthesis, cDNA purification, *in vitro* transcription, aRNA purification, adapter ligation, post-ligation purification, a second 2nd strand synthesis, and purification were carried out using an automated MBS 8000 system, as described previously (Jemt et al., 2016). cDNA was amplified by PCR using Illumina Indexing primers (Stahl et al., 2016) and purified using carboxylic acid beads on an automated MBS robot system (Lundin et al., 2010). An Agilent Bioanalyzer High Sensitivity DNA Kit (Agilent) was used to analyze the size distribution of the final libraries. The concentration of the libraries was measured with Qubit dsDNA HS (Thermofisher). The libraries were sequenced on the Illumina Nextseq platform using paired-end sequencing. Thirty bases were sequenced on read one to determine the spatial barcode and UMI, and 55 bases were sequenced on read two to cover the genetic region. Probes were collected from each well and transferred to separate tubes. Next, 2nd strand synthesis, cDNA purification, in vitro transcription, aRNA purification, adapter ligation, post-ligation purification, a second 2nd strand synthesis and purification were carried out using an automated MBS 8000 system as described previously (Jemt et al., 2016). cDNA was amplified by PCR using Illumina Indexing primers (Stahl et al., 2016) and purified using carboxylic acid beads on an automated MBS robot system (Lundin et al., 2010). An Agilent Bioanalyzer High Sensitivity DNA Kit (Agilent) was used to analyze the size distribution of the final libraries. The concentration of the libraries was measured with Qubit dsDNA HS (Thermofisher).

### 2.7 Staining of the slide features

After the probes were released from the slide surface, the features with remaining non-cleaved DNA probes were detected by incubation with hybridization mixture containing Cyanine-3 labeled oligonucleotides, as described previously (Stahl et al., 2016). Fluorescent images were acquired using the same microscope as for the bright field images.

### 2.8 Sequencing

The libraries were sequenced on the Illumina Nextseq platform using paired-end sequencing. Thirty bases were sequenced on read one to determine the spatial barcode and UMI, and 55 bases were sequenced on read two to cover the genetic region.

### 2.9 Image alignment and spot detection

Bright field-stained images (H&E) and fluorescent images (Cy3) were aligned using the ST Spot Detector (Wong et al., 2018) software. The pixel and respective array coordinates of the detected spot centroids (inside tissue) were exported to a file that was used for down-stream analysis and visualization.

### 2.10 Data processing

Sequenced raw data was processed using the open-source ST Pipeline v1.45 (Navarro et al., 2017) with the genome reference Ensembl GRCm38 v86 and reference Mouse GenCode vM11 (Comprehensive gene annotation). The ST Pipeline was executed with the following settings: Enable homopolymers filter (A, G, T, C, N) with a length of 10, enable two-pass mode for the alignment step, remove non-coding RNA (using the v86 non coding RNA database from Ensembl), discard reads whose UMI has more than 6 low quality bases, and discard trimmed reads shorter than 20.

The matrices of counts (spots by genes) generated by the ST Pipeline were filtered to replace Ensembl IDs by gene names and to keep only protein-coding, long-non-coding-intergenic, and antisense RNAs. The matrices of counts underwent another filtering step where only spots inside the tissue were kept using the file generated in the previous step (image alignment).

### 2.11 Datasets

The Tau22 dataset is composed of 2 sections per animal (mice) and 3 animals per genotype (Tau22 and littermate WT). Likewise, the TauKO dataset is composed of 2 sections per animal (mice) and 3 animals per genotype (TauKO and littermate WT). The two WT strains are not the same since they were continuously bred to their respective genetically modified tau strains. We did not do a direct Tau22 to TauKO analysis.

### 2.12 Data analysis (Tau22)

The filtered and aligned matrices of counts were analyzed jointly with the Scanpy (Wolf et al., 2018) package v1.8.2. Briefly, the spots with a total count (UMIs) less than 2,000 or bigger than 40,000 were discarded. The spots with less than 1,000 RNAs detected (count > 0) or with a percentage of mitochondrial RNAs above 15 were also discarded, mitochondrial RNAs were consequently removed from the filtered data and the remaining RNAs that were detected in less than 10 spots were also discarded. This resulted in 7,403 spots and 12,748 RNAs after filtering. The filtered data was normalized using the *normalize_total* function in Scanpy, the normalized data was log-transformed using a pseudo count of 1. The normalized and log-transformed data was adjusted to remove the unwanted batch effect of the animal (mice) using the *regress_out* function in Scanpy. Using the batch-corrected data, we selected the top 2000 variable RNAs using the “Seurat” flavor implemented in Scanpy. We used the 7,403 spots and the 2000 RNAs to perform unsupervised clustering which consisted in: (1) scale data to unit variance, (2) dimensionality reduction with PCA (Pearson, 1901), (3) compute the k-nearest neighbors (*k* = 15), (4) build a 2D manifold with UMAP (McInnes et al., 2018), and (5) compute clusters with the leiden algorithm (Traag et al., 2019) using a resolution of 0.75. The Allen Brain Atlas (Lein et al., 2007) and the tissue sections (H&E) were used to annotate the clusters. The clustering was validated by looking for technical effects by tissue morphology, count, animal, and genotype.

### 2.13 Data analysis (TauKO)

The filtered and aligned TauKO matrices of counts were analyzed in the same way as the Tau22 dataset with the exception that 6,437 spots and 11,073 RNAs were obtained after filtering, the unsupervised clustering produced 16 well-defined clusters, and no batch effect could be observed. Similarly, to the Tau22 dataset, the Allen Brain Atlas (Lein et al., 2007) and tissue section images (H&E) were used to annotate the clusters.

### 2.14 Spatial differential expression analysis (S-DE)

We used sepal (Andersson and Lundeberg, 2021) to infer RNAs with spatially distinct patterns. Sepal can only be run on individual sections, and it ranks the RNAs by a score. We averaged the scores for all the Tau22 and TauKO sections separately and then selected the top 25 RNAs, respectively. We validated the results by plotting the normalized expression of the RNAs onto the tissue sections.

### 2.15 Region and genotype based differential expression analysis (DE)

We used the diffpy (Fischer and Hölzlwimmer, 2021) package to leverage on the power of its GLM zero inflated negative binomial models to infer RNAs that were differentially expressed for each region of interest. Each strain was compared to its respective littermate WT controls, thus Tau22 vs. WT and TauKO vs. WT were compared for the regions of interest (hippocampus and ventricle). An RNA was considered differentially expressed if the adj *p*-value ≤ 0.05 and a fold change of |0.5| for hippocampus and ventricle regions.

### 2.16 Enrichment analysis

The sets of differentially expressed RNAs were queried for GO biological processes (Ashburner et al., 2000) enriched pathways using the gprofiler (Kolberg et al., 2023) package. Only the terms labelled as “significant” (*p*-value below 0.05) were reported with the corresponding significantly changed RNAs.

### 2.17 Immunofluorescence

Immunofluorescence (IF) on mouse brain sections was performed as described previously (Zheng et al., 2020). Briefly, sagittal (5 μM) brain slices were deparaffinized and unmasked using citrate buffer (12 mM citric acid, 38 mM sodium phosphate dibasic, pH 6) for 8 min in a pressure tank. The slices were submerged for 1 h in 1% goat serum (Vector Laboratories #S-1000), and the primary antibodies were incubated overnight at 4 °C in the presence of PBS-0.2% Triton. Primary antibodies were revealed via secondary antibodies coupled to Alexa 488 or 568 (Life Technologies; 1/1000). The sections were counterstained with 4′,6-Diamidino-2-phenylindole (DAPI) and mounted with fluorescence mounting medium (Agilent Dako #S3023). The following primary antibodies were used: TTR (ThermoFisher Scientific PA5-88094; 1/100), AT8 (PSer202/Thr205tau; ThermoFisher Scientific MN1020; 1/400), Aβ (MOAB2; reactive to Aβ aa 1–4, Novus Biologicals NBP2-13075; 1/500) (4G8; reactive to Aβ aa 17–24; BioLegend SIG-39200; 1/5000). Fluorescence was quantified using the FIDJI macro application of ImageJ (confocal microscopy platform, PBSL, UAR2014/US41, Lille). Quantification corresponds to the z stack of serial confocal sections covering the entire thickness of the brain section. For CP epithelial cells, in each section the cell set was manually delimited and fluorescence was quantified in all cells. For Aβ deposits, in each section, labeled clusters were manually delimited and fluorescence was quantified. The quantification shows the mean of fluorescence values per individual.

Fluorescence from mouse brain sections was acquired using an LSM 710 confocal laser-scanning microscope (clsm) (Carl Zeiss). The confocal microscope was equipped with a 488-nm Argon laser, 561-nm diode-pumped solid-state laser, and a 405-nm ultraviolet laser. The images were acquired using an oil 63X Plan-APOCHROMAT objective (1.4 NA). All recordings were performed using the appropriate sampling frequency (16 bits, 1,024–1,024 images, and a line average of 4).

### 2.18 Statistics for immunofluorescence analysis

The Shapiro–Wilk test of normality (GraphPad Prism 7) was used to test if the data were normally distributed. Two-tailed, unpaired t-test (GraphPad Prism 7) was used for statistical analysis of immunofluorescence in murine brains. Each biological replicate corresponds to one mouse. The number of biological replicates is indicated in the legends. The experimenters were not blinded. Data are presented as mean ± SEM, *p < 0.05; **p < 0.01.

## 3 Results

### 3.1 ST identifies DE RNAs and molecular clusters corresponding to anatomical layers of the mouse hippocampus and ventricles

**Figure 1:**
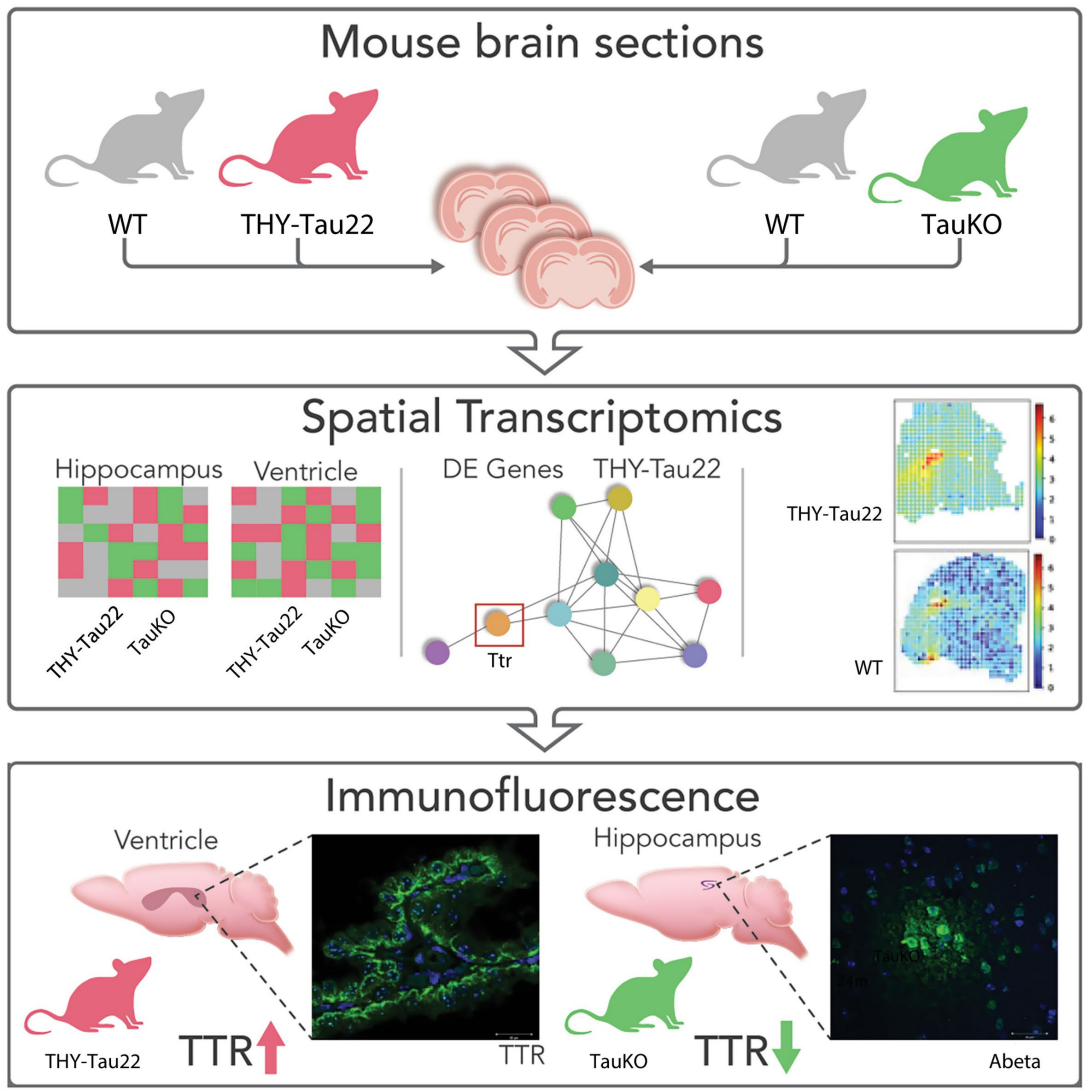
Graphic representation of data. **(A)** UMAP plot of Tau22 dataset. **(B)** UMAP plot highlighting the regions used in the analysis of the Tau22 mouse regions. **(C)** Venn diagram depicting DE RNAs from the hippocampus and ventricles of Tau22 mice. **(D)** UMAP plot of TauKO dataset. **(E)** UMAP plot highlighting the spots used in the analysis of the TauKO mouse regions. **(F)** Venn diagram depicting DE RNAs from the hippocampus and ventricles of TauKO mice. Genes depicted above/below numbers in C and F denote upregulated or downregulated genes Tau22 or TauKO vs. WT, respectively.

A graphic overview of our experimental approach is shown in Figure 1. Briefly, snap frozen tissue samples from three animals of each genotype (Tau22, TauKO, and their respective littermate WT controls), were cryo-sectioned and processed as described in the Methods. Only female mice were used in this study. The Tau22 dataset is composed of 2 sections per animal (mice) and 3 animals per genotype (Tau22 and WT), the total number of spots under the tissue is 7,483 with 22,174 RNAs. Box plots showing total read counts and number of detected RNAs per spot are shown in Supplementary Figure S1. The average number of reads (UMIs) per spot is 13,784 with an average of 4,797 detected RNAs per spot (Supplementary Figures S1A,B). Similarly, we obtained 6,857 spots and 21,974 unique RNAs for the TauKO dataset with an average number of UMI reads per spot of 6,714 and 3,116 detected RNAs per spot (Supplementary Figures S1C,D).

Clustering analysis was performed for each dataset (see Methods). Characterization of Tau22 mice data is shown in Supplementary Figure S2 and TauKO data is shown in Supplementary Figure S3. UMAP manifolds derived from the cluster analysis were colored by genotype, animal, and total expression to test for the presence of batch effects and to assess the robustness of the clustering (Supplementary Figures S2A, S3A). In all cases, the RNA expression clusters corresponded well to the anatomical layers in the brain hemisphere (Supplementary Figures S2B,C, S3B,C). The H&E-stained tissue sections are shown in panel Supplementary Figure S2B for Tau22 and Supplementary Figure S3B for TauKO, while the clusters for each mouse overlayed on the sections are shown in Supplementary Figure S2C for Tau22 and Supplementary Figure S3C for TauKO. The annotation of the clusters was performed using the H&E images and the Allen Brain Atlas (Lein et al., 2007). We selected, as mentioned previously, the clusters corresponding to the hippocampus and ventricle regions for further analysis. We validated the clustering results by plotting the normalized expression of the RNAs onto the tissue sections (Tau22, Supplementary Figure S2C; TauKO, Supplementary Figure S3C).

**Figure 2:**
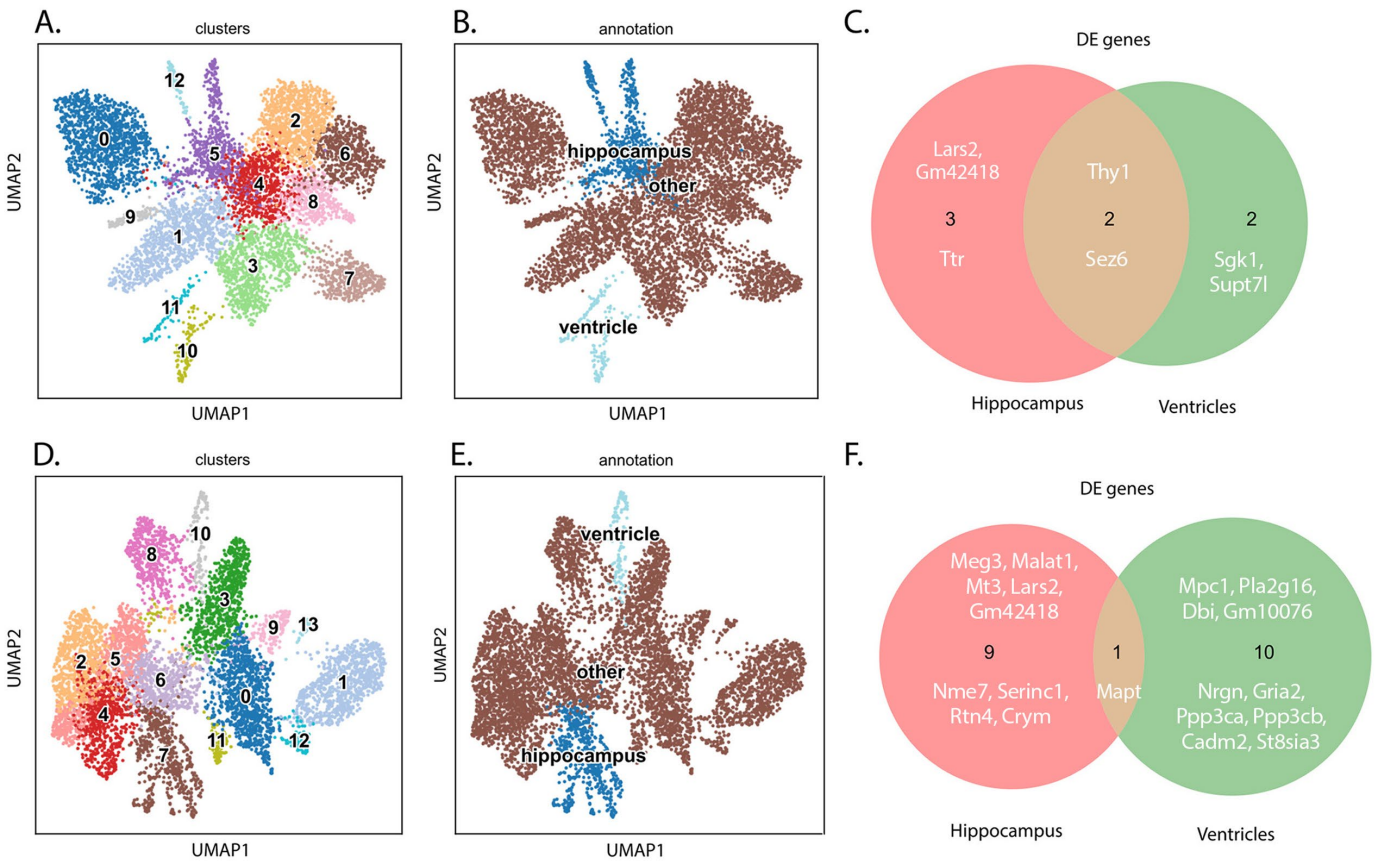
Schematic summary of ST and IF results focusing on TTR expressions. Graphic representation of the study. Two mouse models representative of tau pathology (THY-Tau22) and a tau null (TauKO) were used in a spatial transcriptomics analysis of brain tissue. *TTR* was identified as a DE RNA, its expression was validated by immunofluorescence, and decreased TTR was associated with increased Aβ deposition in TauKO mice.

We elected to focus our attention on the hippocampus and ventricles because tau mice have hippocampus-dependent defects (Schindowski et al., 2006) and the CP dysfunction was recently reported as a newly defined subgroup of AD (Tijms et al., 2024). The clustering analysis was followed by a differential expression (DE) analysis to identify DE RNAs between Tau22-WT (Figures 2A–C) and TauKO-WT (Figures 2D–F) in the regions of interest (hippocampus and ventricles). As noted in the methods, each strain has their own WT mouse littermates. After applying a significance cut off adj. *p*-value of ≤ 0.05 and a fold change of |0.5| on the Tau22 datasets, we obtained 5 DE in the hippocampus (*Thy1, Sez6, Ttr, Gm42418, Lars2*) and 4 RNAs that were DE in the ventricles (*Thy1, Sez6, Sgk1, Supt7l*). Two RNAs were shared between the two regions, *Thy1* and *Sez6*. *Thy1* is a cell surface glycoprotein that functions in cell-to-cell communication (Hu et al., 2022) and *Sez6* encodes a protein altered in Alzheimer’s disease patient’s cerebral spinal fluid and important in neuronal signaling (Munro et al., 2016). For TauKO, there were 10 RNAs that were DE in the hippocampus and 11 RNAs that were DE in the ventricles, and 1 RNA was found in both regions, *Mapt* (Figures 2C,F). The following genes were significantly changed in the hippocampus: *Mapt, Meg3, Serinc1, Nme7, Rtn4, Malat1, Mt3, Crym, Lars2, Gm42418*. In the ventricles of TauKO mice, *Mapt, Nrgn, Mpc1, Gria2, Ppp3cb, Pla2g16, St8sia3, Gm10076, Dbi, Cadm2, and Ppp3ca* were found to be significantly changes. A full list of DE genes per region is listed in Supplementary Table 1.

RNA enrichment analysis using Gene Ontology biological process revealed a single term changed in Tau22 mice, in the hippocampus the term negative regulation of neuron projection development was identified, and the DE RNAs found in the term were *Sez6* and *Thy1*. In contrast, several terms associated with axon extension and projection, and gliogenesis were identified in the TauKO hippocampus (Supplementary Figure S4A). Across the hippocampus terms, three significantly changed RNAs were identified: tau (*Mapt*), metallothionein 3 (*Mt3*), and reticulon-4 (*Rtn4*) (Supplementary Figure S4B). Metallothionein 3 binds heavy metals like zinc and copper, has antioxidant properties, and is downregulated in AD brains (Vasak and Meloni, 2017). Reticulon-4 is a potent neurite outgrowth inhibitor and is thought to promote neuroinflammation and neurodegeneration in AD (Kulczynska-Przybik et al., 2021). The term learning or memory and several terms related to synaptic signaling terms were enriched in the TauKO ventricles dataset (Supplementary Figure S4C). There were five RNAs from TauKO ventricle in the terms, *Gria2*, *Ppp3ca*, *Ppp3cb*, *Nrgn,* and *Mapt* and most of the terms involved synaptic signaling.

In a separate analysis which included all regions (Supplementary Figure S5), we aimed to detect RNAs that had region-wise distinct spatial patterns, in other words RNAs that were spatially and differentially expressed with regards to the other regions (S-DE). To begin, we identified the top DE RNAs from each genotype comparison (Supplementary Figures S5A,C). Next, we identified the top DE RNAs that also displayed a spatial pattern. The top DE and S-DE RNAs are shown in the heatmaps per genotype and region (Supplementary Figures S5A,B, Tau22 versus its WT, Supplementary Figures S5C,D, TauKO versus its WT). Notably, *Ttr* was both a top DE RNA and top S-DE RNA in the Tau22 dataset. It was not in the TauKO significantly changed RNA list. Additionally, we found minimal overlap, two RNAs *Lars2* and *Gm42418*, among the RNAs in the hippocampus datasets in the Tau22 and TauKO comparisons by region and no shared genes across the ventricle’s datasets.

### 3.2 Tau and pathological forms of tau may regulate TTR protein levels in CP epithelial cells

**Figure 3:**
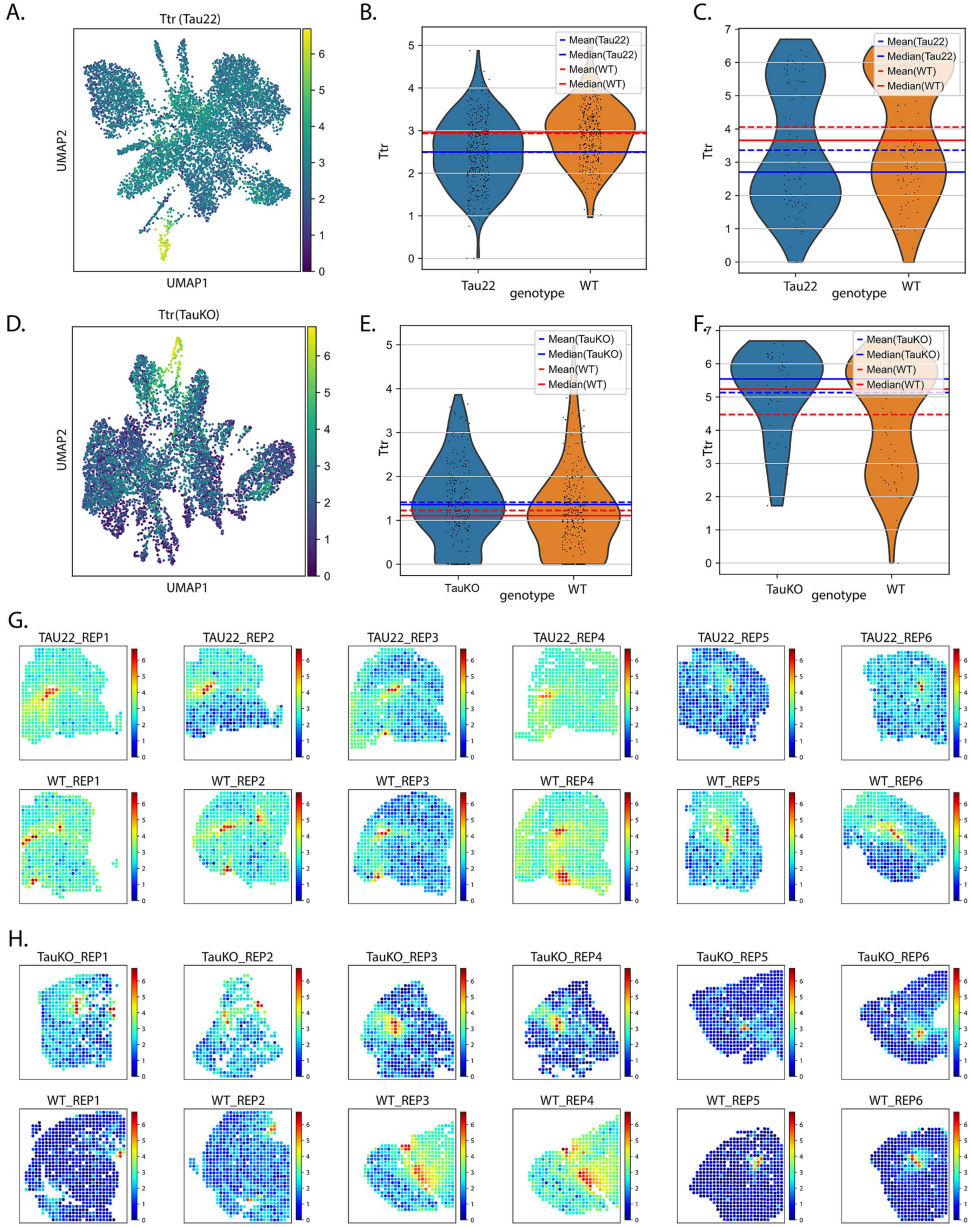
Investigation of *Ttr* RNA expression. **(A)** UMAP plot showing the expression pattern of *Ttr* RNA in Tau22 mice. Violin plots of data *Ttr* expression data from the hippocampus **(B)** and ventricles **(C)** of Tau22 mice. **(D)** UMAP plot showing the expression pattern of *Ttr* RNA in TauKO mice. Violin plots of data *Ttr* expression data from the hippocampus **(E)** and ventricles **(F)** of TauKO mice. **(G)** *Ttr* RNA expression plotted onto the tissue sections of Tau22 mice. **(H)** *Ttr* RNA expression plotted onto the tissue sections of TauKO mice.

The ST results revealed a number of RNAs whose transcript expression were dysregulated in the presence of tau pathology and/or *tau* (*Mapt*) deletion in the hippocampus and the lateral ventricles (Figure 3). Among the RNA lists, we noted the RNA *Ttr.* We elected to focus on Ttr because of its known neuroprotective properties, it is important for memory, and plays a role in Aβ sequestration and clearance (Brouillette and Quirion, 2008; Buxbaum, 2023; Buxbaum and Johansson, 2017; Buxbaum et al., 2008; Cascella et al., 2013; Ciccone et al., 2020; Costa et al., 2008; Gomes et al., 2016; Gonzalez-Marrero et al., 2015; Iqbal, 2018; Li et al., 2013; Liz et al., 2020; Nilsson et al., 2018; Santos et al., 2010; Schwarzman et al., 1994; Ueda, 2022; West et al., 2021). Further, *Ttr* was a top RNA in both the S-DE and DE analysis in the Tau22 data set (Supplementary Figures S5A,B). Figures 3A–C displays the expression pattern and transcript levels of *Ttr* from Tau22 mice from the hippocampus and ventricles, whereas Figures 3D–F show similar results from the TauKO mice. The plots show the mean and median values to facilitate easier comparison. The RNA plots of *Ttr* expression are overlaid on the tissue slices in Figure 3G for Tau22 mice and Figure 3H for TauKO mice. *Ttr* is an RNA coding for an extracellular chaperone deregulated in AD but there is little known about a regulatory relationship between tau and TTR protein.

**Figure 4:**
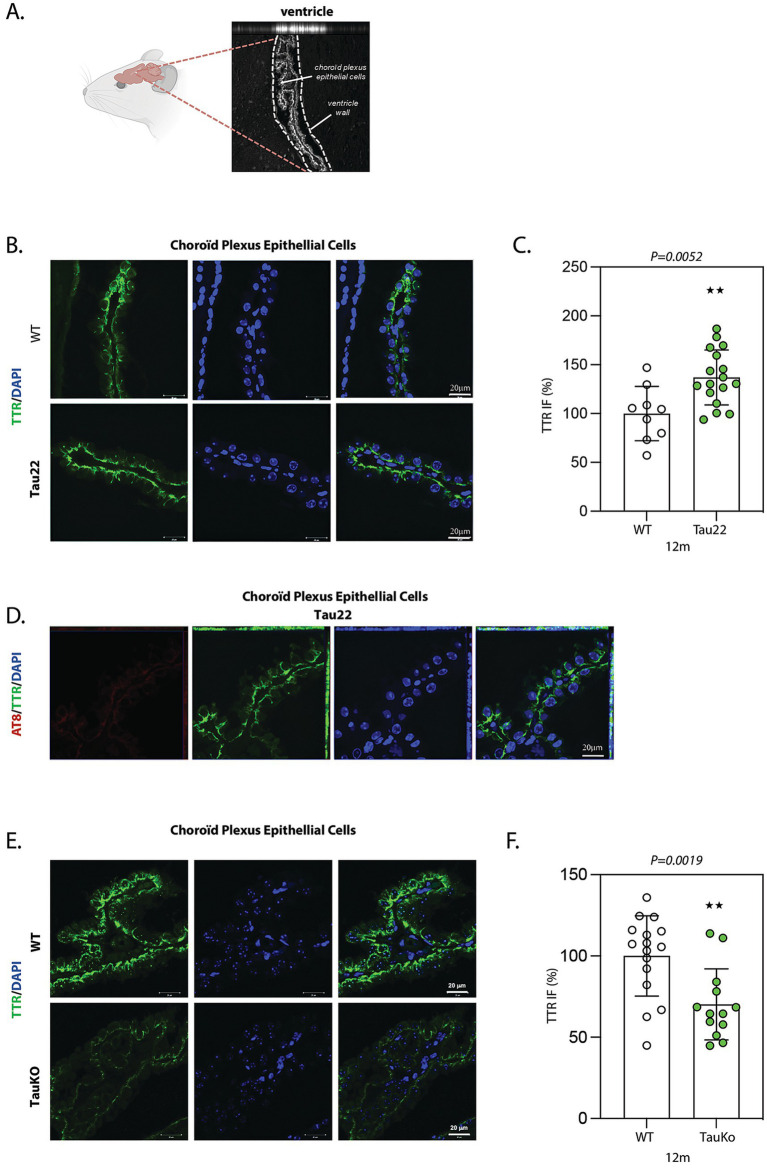
TTR is increased in choroid plexus epithelial cells from Tau22 mouse brains. **(A)** Representative image of ventricles in sagittal sections from 12 months old WT mouse brain. Choroïd plexus (CP) epithelial cells present inside the ventricles are labeled with an anti-TTR antibody. **(B)** Representative images of sagittal sections from 12 months old WT (*n* = 9) and Tau22 (*n* = 17) mouse brains. The sections were labeled with the anti-TTR antibody. IF signals were analyzed by clsm (z projection). Nuclei were detected with DAPI staining. The scale bars represent 20 μm. **(C)** The intensity of the TTR IF signals were quantified within CP epithelial cells from 12 months old WT and Tau22 mouse brains. Graph shows the mean of TTR fluorescence per mouse. Each biological replicate represents one mouse. Data are presented as mean ± SEM (**p < 0.01; Mann Whitney U test). **(D)** Representative images of sagittal sections from 12 months old Tau22 (*n* = 17) mouse brains. The sections were labeled with the phospho-dependent anti-tau AT8 and the anti-TTR antibody. IF signals were analyzed by clsm (z projection). Nuclei were detected with DAPI staining. The scale bars represent 20 μm. **(E)** Representative images of sagittal sections from 12 months old WT (*n* = 15) and TauKO (*n* = 13) mouse brains. The sections were labeled with the anti-TTR antibody. IF signals were analyzed by clsm (z projection). Nuclei were detected with DAPI staining. The scale bars represent 20 μm. **(F)** The intensity of the TTR IF signals were quantified within CP epithelial cells from 12 months old WT (*n* = 15) and TauKO (*n* = 13) mouse brains. Graph shows the mean of TTR fluorescence per mouse. Each biological replicate represents one mouse. Data are presented as mean ± SEM (**p < 0.01; Mann Whitney U test).

TTR protein localization and levels were analyzed by IF in sagittal sections from 12 months-old THY-Tau22 and WT littermate mouse brains using TTR antibody. In both genotypes, TTR protein was only strongly detected in CP epithelial cells, where it is secreted (Stauder et al., 1986), with an increasing intensity towards the basal surface (Figures 4A,B). IF analysis revealed a significant increase of TTR protein level in CP epithelial cells from Tau22 mice versus WT littermates (mean intensity WT: 106.6%; Tau22: 130.6%) (Figures 4B,C). We did not observe accumulation of phosphorylated tau in CP epithelial cells (Figure 4D), indicating the absence of tau pathology there.

We also evaluated the phosphorylated form of tau (ptau) and TTR expression in the hippocampus of WT and Tau22 mice, Supplementary Figures S6A–E, respectively. Although increased levels of TTR protein have been previously reported in hippocampal cells from AD-like transgenic mouse models (Li et al., 2011; Stein and Johnson, 2002), no difference in the level of TTR was observed in CA1 hippocampal cells between 12 months-old WT and Tau22 mouse brains (Supplementary Figures S6D,E). Additionally, no TTR expression was detected in neurons displaying hyperphosphorylated tau (Supplementary Figure S6C).

To better understand whether the elevation of TTR observed in Tau22 mice associates with a gain or a loss of tau function, we further explored the influence of tau deletion on TTR expression by IF in sagittal sections from 12 months old TauKO and WT littermate mouse brains using TTR antibody. Again, in WT mice, high TTR expression was restricted to CP epithelial cells from ventricles (Figure 4E). Quantification of TTR IF revealed a statistically significant decrease of TTR in TauKO compared to WT CP epithelial cells (mean intensity WT: 106.4%; TauKO: 64.44%) (Figure 4F). We also evaluated TTR levels in the hippocampus of TauKO mice, and similar levels of TTR were observed in TauKO and WT littermates (Supplementary Figures S6F,G). Our results indicate that tau may positively modulate the level of TTR protein in the CP epithelial cells.

### 3.3 Tau deletion favors Aβ deposits in aged mouse brains

**Figure 5:**
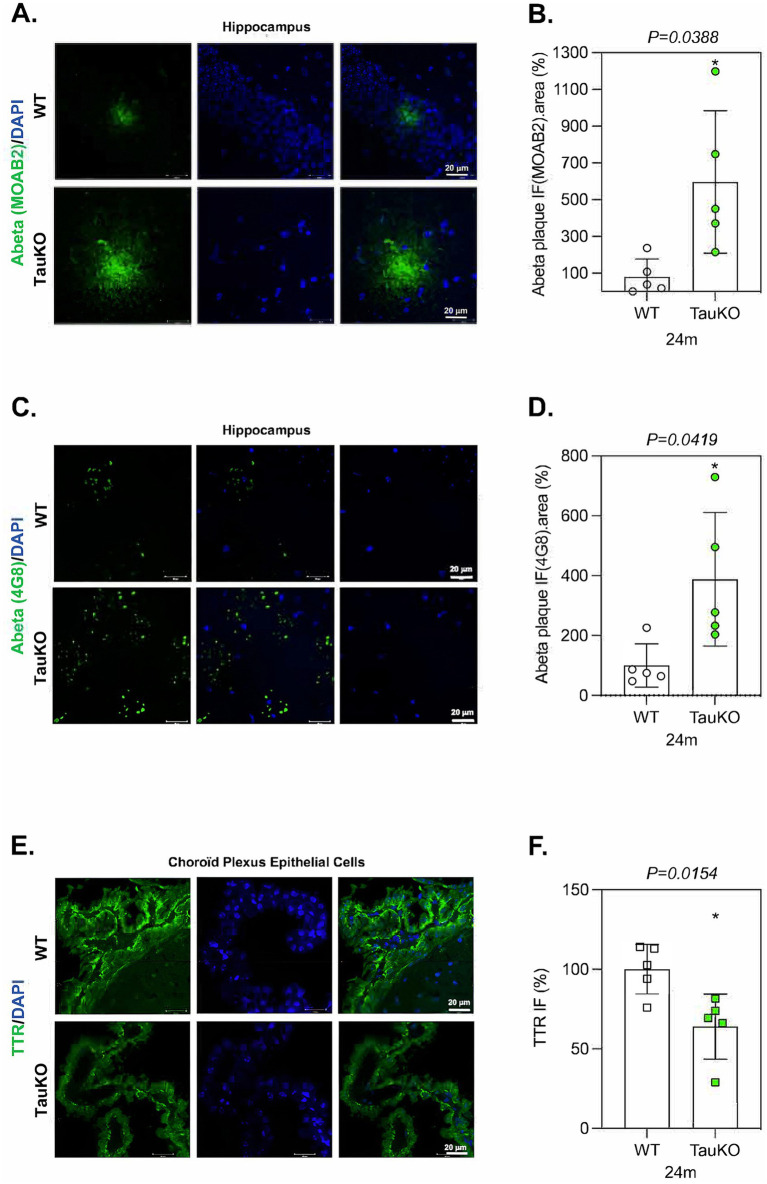
Tau deletion enhances Aβ deposits in aged mouse brains. **(A)** Representative images of sagittal sections from 24 months old WT (*n* = 5) and TauKO (*n* = 5) mouse brains. The sections were labeled with the anti-Aβ antibody MOAB2. IF signals were analyzed by clsm (z projection). Nuclei were detected with DAPI staining. The scale bars represent 20 μm. **(B)** Quantification of IF intensity and the area of Aβ deposits from 24 months old WT (*n* = 5) and TauKO (*n* = 5) mouse brains. Graph shows the mean of Aβ IF.area per mouse. Each biological replicate represents one mouse. Data are presented as mean ± SEM (*p < 0.05; Mann Whitney U test). **(C)** Representative images of sagittal sections from 24 months old WT (n = 5) and TauKO (*n* = 5) mouse brains. The sections were labeled with the anti-Aβ antibody 4G8. IF signals were analyzed by clsm (z projection). Nuclei were detected with DAPI staining. The scale bars represent 20 μm. **(D)** Quantification of IF intensity and the area of Aβ plaques from 24 months old WT (*n* = 5) and TauKO (*n* = 5) mouse brains. Graph shows the mean of Aβ IF.area per category. Each biological replicate represents one mouse. Data are presented as mean ± SEM (*p < 0.05; Mann Whitney U test). **(E)** Representative images of sagittal sections from 24 months old WT (*n* = 5) and TauKO (*n* = 5) mouse brains. The sections were labeled with the anti-TTR antibody. IF signals were analyzed by clsm (z projection). Nuclei were detected with DAPI staining. The scale bars represent 20 μm. **(F)** The intensity of the TTR IF signals were quantified within CP epithelial cells from 24 months old WT (*n* = 5) and TauKO (*n* = 5) mouse brains. Graph shows the mean of TTR fluorescence per mouse. Each biological replicate represents one mouse. Data are presented as mean ± SEM (*p < 0.05; Mann Whitney U test).

TTR synthesized in CP epithelial cells is secreted into the CSF that bathes the whole brain. In transgenic Tg2576 mice, an AD-like mouse model that expresses mutant APP(sw), Aβ peptide is overexpressed and TTR expression increases in hippocampal neurons likely as a compensatory mechanism to prevent Aβ aggregation in the brain (Li and Buxbaum, 2011; Stein and Johnson, 2002). Since TTR may prevent Aβ peptide aggregation, we hypothesized that tau deletion in aged mice might induce the formation of Aβ amyloid deposits. To test this proposition, we compared the effect of tau deletion-induced TTR downregulation on the presence of Aβ deposits in sagittal sections from 12 and 24 months-old TauKO and WT littermate mouse brains using the Aβ antibody MOAB2. Although only few Aβ deposits were detected both in 12 months-old TauKO and WT littermate mouse brains (Supplementary Figure S7), a strong increase of the area and IF intensity of Aβ aggregates was observed in the hippocampus and/or near the lateral ventricles of 24 months-old TauKO compared to WT mouse brains (mean intensity WT: 100%; TauKO: 596.3%) (Figures 5A,B). Similar results were obtained using a second anti-Aβ antibody (4G8, mean intensity WT: 100%; TauKO: 387.9%) (Figures 5C,D). It is noticed that the aspect of the plaques detected by MOAB2 and 4G8 is different and potentially linked to the distinct epitopes recognized by the two antibodies. Nevertheless, it should be stressed that the use of 4G8 to detect murine Aβ is controversial. No Aβ accumulation was observed in CP epithelial cells of 24 months-old TauKO compared to WT mouse brains (data not shown).

For comparison, we also evaluated TTR protein level, and it was downregulated in CP epithelial cells from 24 months-old TauKO mouse brains (mean intensity WT: 102.7%; TauKO: 69.42%) (Figures 5E,F) in a similar range as previously observed in 12 months-old TauKO mice (Figures 4E,F), showing that the regulatory role of tau on TTR protein expression is conserved during aging. Altogether these results show that tau deletion potentiates Aβ deposit formation of endogenous Aβ peptide in aged mice, and that this correlates with a decrease in TTR expression.

### 3.4 Regulation of TTR by tau

**Figure 6:**
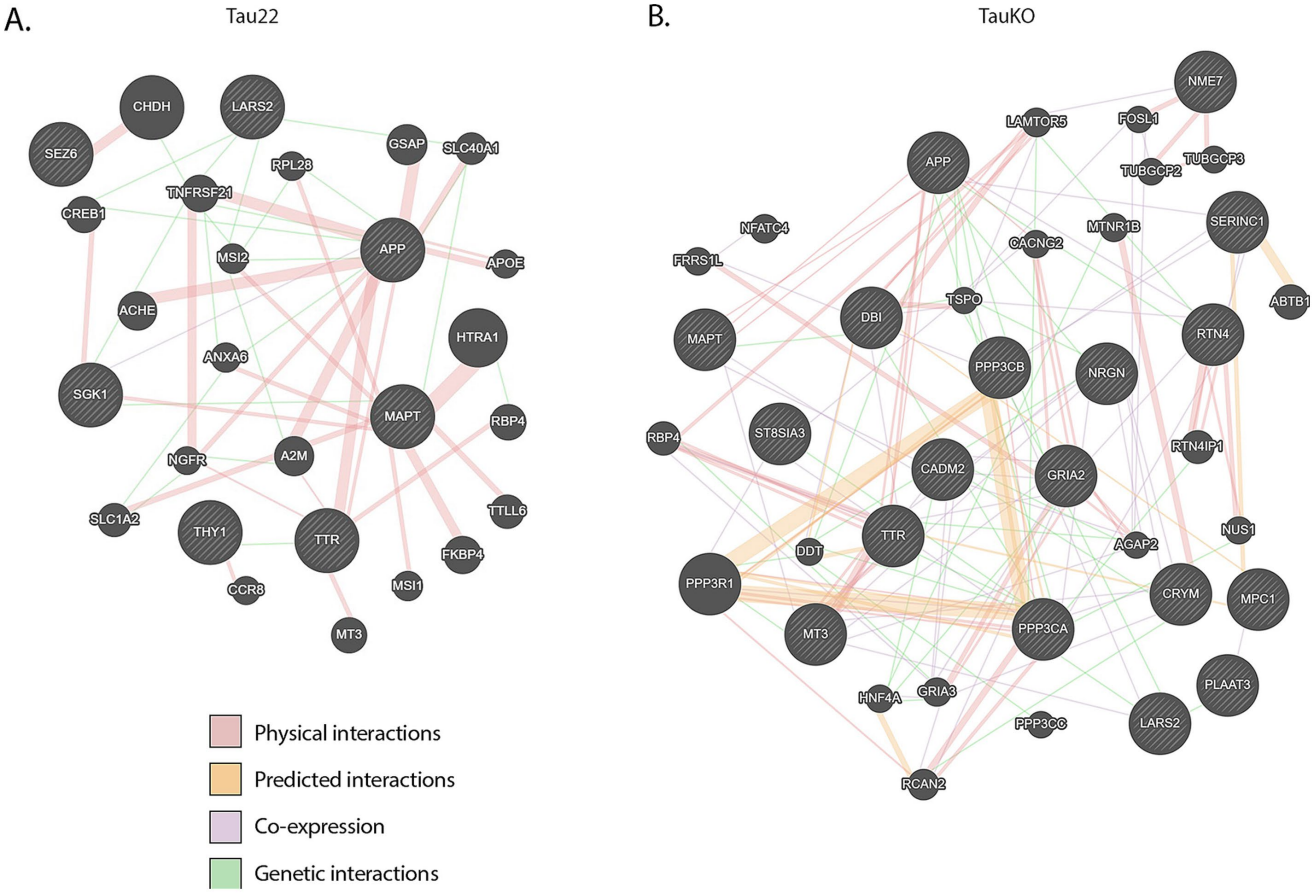
GeneMANIA networks. **(A)** Graphic representation of Mapt, APP (Aβ), TTR, and both DE gene lists from Tau22 mice. **(B)** Graphic representation of Mapt, APP (Ab), TTR, and both DE gene lists from TauKO mice.

To explore the interconnectedness between tau (*Mapt*), APP (presumably Aβ), and TTR, we submitted those genes plus our DE gene lists, by genotype, to GeneMANIA (Warde-Farley et al., 2010). The output graphics are shown in Figure 6. There were no known direct linkages between tau and TTR, neither physical nor predicted. No transcription factors were revealed by this analysis either. However, there was an indirect link between tau, APP (Aβ), and TTR. Stringdb analysis produced similar results (data not shown). It appears that the shortest connection between Ttr and tau is through Aβ.

## 4 Discussion

Collectively, ST and IF reveal a new regulatory role of tau on the expression of TTR. In connection with these findings, this study highlights the key protective role of murine tau on endogenous Aβ deposition in mouse brain. Our data provides novel insight into the physiological role of tau in regulating TTR protein level in CP epithelial cells and the gain of this function associated with tau pathology.

We should note that while this paper was in preparation, Ali et al. (2024) reported single-cell analysis results performed on the cortex of 7-month-old Tau22 and wild type littermate mice, an early time point of pathological development in the cortex of THY-Tau22 mice. *Ttr* RNA expression was increased in Tau22 in neurons in multiple cell types, astrocytes, microglial cells, and oligodendrocytes. This goes in the same direction as our IF results in 12-month-old Tau22 mice. Together, this reinforces the correlation between the development of pathological forms of tau and altered expression of TTR in the mouse brain.

Proteins synthetized in CP epithelial cells are directly secreted into the CSF, which bathes the entire brain. Therefore, any variation in the expression of secreted proteins from CP epithelial cells can have important consequences throughout the brain. Changes in the TTR level in CP epithelial cells of tau transgenic mice likely leads to alteration of TTR in the CSF. Notably, clinical studies in humans have shown that TTR levels in CSF may vary with the stage of pathology (Buxbaum, 2023). In a recent paper by Tijms et al. (2024), using mass spectrometry proteomics in human cerebrospinal fluid from 187 controls and 419 AD patients, the authors defined five AD molecular subtypes including a new one related to CP dysfunction. Importantly, our results suggest that the contribution of CP dysfunction, in a subgroup of AD patients, may be linked to tau pathology. We should note that while it is tempting to speculate that our results may pertain to human disease, further analysis is warranted to validate whether this is so. Nonetheless, the relationship between CP dysfunction, TTR expression, and tau pathology in AD warrants further investigation.

It has been previously shown that tau deletion increases Aβ plaques when human Aβ is overexpressed in mouse brain (Lonskaya et al., 2014), but ours is the first description that reports tau deletion potentiates endogenous mouse Aβ deposition. Interestingly we show decreased TTR levels in CP epithelial cells from tau-deleted mice, and this correlates with an accumulation of Aβ deposits in the hippocampus of aged mice. Our results suggest that a reduction of TTR, induced by the loss of tau, may participate to promote accumulation of Aβ peptide in the brain. However, the involvement of murine TTR in murine Aβ aggregation requires further investigation. Furthermore, different approaches such as Western blot or ELISA would be needed to clarify which Aβ form (monomeric, oligomeric or fibrillar) was identified by IF.

Here, we propose that late tau pathology of the transgenic mouse model THY-Tau22 promotes increased TTR protein expression, indicating a gain of tau function. Besides Aβ, TTR can prevent the amyloidogenesis of various unstructured proteins (Magalhaes et al., 2021; West et al., 2021). Notably, tau is also an intrinsically unstructured protein which can form intraneuronal amyloid fibrils and is present with high molecular weight species in the brain interstitial fluid (ISF) in pathological conditions (Takeda et al., 2015), and the ability of TTR to inhibit tau aggregation has not been mentioned in the literature. Here we observe that in response to late tau pathology, TTR protein level is increased in CP epithelial cells from Tau22 mouse brains. Surprisingly, tau aggregation peaks at 12 months old in Tau22 mouse brains and no longer progresses as mice age suggesting that mechanisms are induced to block the progression of the pathology (Schindowski et al., 2006). Therefore, it is tempting to speculate that increasing the amount of TTR in the CP epithelial cells of Tau22 mouse brain is a backup mechanism to slow down amyloid protein aggregation processes including tau. It would be worth testing this hypothesis and establish if TTR can directly bind to tau and inhibit its aggregation process, but that is beyond the scope of this work.

Although CP epithelium cell failure is described as an early sign in the etiology of AD (Giao et al., 2022), potential alteration of TTR levels in the CSF of AD patients is still controversial (Bergen et al., 2015; Riisoen, 1988; Serot et al., 1997; Tijms et al., 2024). Raha-Chowdhury et al. (2019) suggested that the presence of insoluble phosphorylated tau in CP epithelial cells from AD brains may favor Aβ aggregation. Conversely, in our transgenic mouse model Tau22 where pathological forms of tau are not detected in the CP, results show no effect of tau pathology on Aβ agglomeration in the brain. Of course, we cannot exclude that in late phases of AD, when they invade the CP, insoluble forms of tau disrupt the functionality of CP epithelial cells, and particularly the synthesis of TTR, thus promoting Aβ peptide aggregation. In view of our results, it is important to unambiguously evaluate the level of TTR in CP epithelial cells and in the CSF of AD and other tauopathies patients.

Previously, genome wide analysis demonstrated that nuclear tau could bind to a fraction of genic protein-coding DNA sequences in neurons (Benhelli-Mokrani et al., 2018). Tau was not found to bind to the DNA of *Ttr* RNA based on the ChIP-on-chip results in neurons (Benhelli-Mokrani et al., 2018). However, regulation of *Ttr* transcription may vary according to cell type (hepatocytes, CP epithelial cells, or neurons) (Costa et al., 1988; Dickson et al., 1986; Wang et al., 2014). Nevertheless, the absence of detection of phosphorylated tau in the CP epithelial cells of Tau22 mice suggests that the effect is likely not direct. To further investigate the relationship between tau, Aβ, and TTR, we constructed GeneMANIA interaction networks with those genes plus the DE genes (Figure 6). No transcription factors or obvious candidate proteins were seen in these graphics to explain how changes in tau modulates the transcription or protein levels of TTR in the brain. However, we note that we have long non-coding RNA (lncRNA) in our DE gene lists and lncRNAs can modulate transcription and act at a distance via transport in extracellular vesicles. Using human iPSC-neurons with Mapt variants, Bhagat et al. (2023) found that the Mapt variants also showed altered expression of Malat1 and Meg3. Further, Malat1 is altered in AD patient plasma and CSF (Zhuang et al., 2020) and has been found in glioma stem cell-derived extracellular vesicles (Yang et al., 2019). We have no knowledge whether Gm42418, Malat1, or Meg3 can alter TTR protein expression. This warrants further investigation.

In addition, a surprising point in this study is that, although the level of the TTR protein is markedly reduced in CP epithelial cells of tau-deleted mice, the dysregulation of the expression of the *Ttr* RNA in the ventricles is not apparent from the ST analysis. Either there was a technical limitation in the sensitivity of detection of mRNAs at the level of CP epithelial cells or something masked the effect of tau on the transcription of the *Ttr* RNA, or the regulatory role of tau is not at the transcriptional level. Tau could modulate the translation, transport, or the degradation of TTR. Indeed, the molecular mechanisms underlying the changes in tau-dependent TTR protein expression in CP epithelial cells remain uncovered.

Our results open new perspectives on the regulation of TTR by tau expression in the brain and for the first time link TTR and tau in neuronal functioning and TTR dysregulation in the context of tauopathies, see Figure 1 for a graphic summation of the study. The ramifications of the associations between tau, TTR, and Aβ warrant further investigation regarding Aβ clearance and cognition.

## 5 Limitations of the study

ST is a formidable technique for obtaining information that is inaccessible by global transcriptional analysis approaches, the fact remains that it still has limitations. This study was initiated when the size of the array spots was 100 μm, which is much larger than current ST methodologies (10-50x). We believe, however, that the 1 K arrays still provide valuable and powerful data.

We recognize that the experiments were conducted using only 12 m and 24 m old mice. Since disease progression is not static, and insights may be missed by snapshot analyses, in future studies we suggest including multiple time points (e.g., 3, 6, 18, or 24 months) which could provide a more comprehensive understanding of the temporal dynamics of the tau-TTR-Aβ relationships, but this is beyond the scope of this paper.” Further, this option was not available to us because of limited resources.

Another limitation of this study is the use of females exclusively. We recognize that we may have missed sex-based differences since we did not use male mice and further that our findings may not entirely translation to males. Further, since our findings are in mice, and while it is tempting to speculate that our results may pertain to human disease, further analysis is warranted to validate whether this is so.”

We also acknowledge that the use of 4G8 to detect murine Αβ is controversial. However, it has been used previously in APOE transgenic mice to detect mouse Aβ (Ding et al., 2008). Further, the most abundant pattern identified by 4G8 is F-x-A (Baghallab et al., 2018) which is highly conserved across species including in the mouse Aβ (Flemmig et al., 2018).

## Data Availability

All the data and images used in the analyses are available at Mendeley Data (10.17632/myby6tfnh7.1). The code used in the analysis and to generate the figures is available at: https://github.com/jfnavarro/TAU22_KOTAU.

## Supplementary material

SUPPLEMENTARY FIGURE S1 Quality control for of the ST analysis. **(A)** Average number of counts per spot in each Tau22 mouse. **(B)** Average number of RNAs detected per spot in each Tau22 mouse. **(C)** Average number of counts per spot in each TauKO mouse. **(D)** Average number of RNAs detected per spot in each TauKO mouse.

SUPPLEMENTARY FIGURE S2 Graphic representation of Tau22 mouse data. **(A)** UMAP plots colored by total number of counts per total RNAs detected, per mouse, and genotype. **(B)** H&E-stained brain sections for Tau22 mice. **(C)** Spatial representation of spots colored by cluster in Tau22 mouse brains.

SUPPLEMENTARY FIGURE S3 **(A)** UMAP plots colored by total number of counts per total RNAs detected, per mouse and genotype. **(B)** H&E-stained brain sections for TauKO mice. **(C)** Spatial representation of spots colored by cluster in TauKO mouse brains.

SUPPLEMENTARY FIGURE S4 RNA ontology analysis. **(A)** RNA set enrichment analysis (GSEA) of the top GO biological process terms from TauKO hippocampus dataset. **(B)** Heatmap of the significantly changed DE RNAs found in various terms. **(C)** GSEA of the top GO biological process terms from TauKO ventricles dataset.

SUPPLEMENTARY FIGURE S5 Heatmaps of top Spatial DE and DE RNAs. **(A)** Top DE RNAs from Tau22 mice. **(B)** Intersection of top spatial DE and DE RNAs from Tau22 mice. **(C)** Top DE RNAs from TauKO. **(D)** Intersection of top spatial DE and DE RNAs from TauKO mice.

SUPPLEMENTARY FIGURE S6 Tau alterations do not affect TTR expression in hippocampal cells from Tau22 mouse brains. **(A)** Schematic representation of the hippocampus from mouse brain. **(B)** Representative image of sagittal sections from 12 months-old Tau22 mouse hippocampus. The section was labeled with the phospho-dependent anti-tau AT8 antibody. IF signals were analyzed by clsm (z projection). Nuclei were detected with DAPI staining. The scale bar represents 200 μm. **(C)** Representative images of sagittal sections from Tau22 (*n* = 12) mouse brains. The sections were labeled with the phospho-dependent anti-tau AT8 and the anti-TTR antibody. IF signals were analyzed by clsm (z projection). Nuclei were detected with DAPI staining. The scale bars represent 50 μm. **(D)** Representative images of sagittal sections from WT (*n* = 13) and Tau22 (*n* = 12) mouse brains. The sections were labeled with the anti-TTR antibody. IF signals were analyzed by clsm (z projection). Nuclei were detected with DAPI staining. The scale bars represent 50 μm. **(E)** The intensity of the TTR IF signals were quantified within hippocampal CA1 cells from WT (*n* = 13) and Tau22 (*n* = 12) mouse brains. Graph shows the mean of TTR fluorescence per genotype. Each biological replicate represents one mouse. Data are presented as mean ± SEM (ns: *p* > 0.05; Mann Whitney U test). **(F)** Representative images of sagittal sections from 12 months-old WT (*n* = 15) and TauKO (*n* = 13) mouse brains. The sections were labeled with the anti-TTR antibody. IF signals were analyzed by clsm (z projection). Nuclei were detected with DAPI staining. The scale bars represent 50 μm. **(G)** The intensity of the TTR IF signals were quantified within hippocampal CA1 cells from WT (*n* = 15) and TauKO (*n* = 13) mouse brains. Graph shows the mean of TTR fluorescence per category. Each biological replicate represents one mouse. Data are presented as mean ± SEM (ns: *p* > 0.05; Mann Whitney U test).

SUPPLEMENTARY FIGURE S7 Aβ deposits are only weakly detected in 12 months old Tau22 and WT littermate mouse brains. Representative images of sagittal sections from 12 months old WT (*n* = 15) and TauKO (*n* = 13) mouse brains. The sections were labeled with the anti-Aβ antibody MOAB2. IF signals were analyzed by clsm (z projection). Nuclei were detected with DAPI staining. The scale bars represent 50 μm.

SUPPLEMENTARY Table 1 List of DE genes per region.
