# Supplementary figures and images for: Spatial transcriptomics reveals an unexpected impact of tau and tau pathology on the expression of transthyretin
Source: Front Aging Neurosci. 2025 Oct 31;17:1656850. doi: 10.3389/fnagi.2025.1656850 (PMC12615506; doi:10.3389/fnagi.2025.1656850)

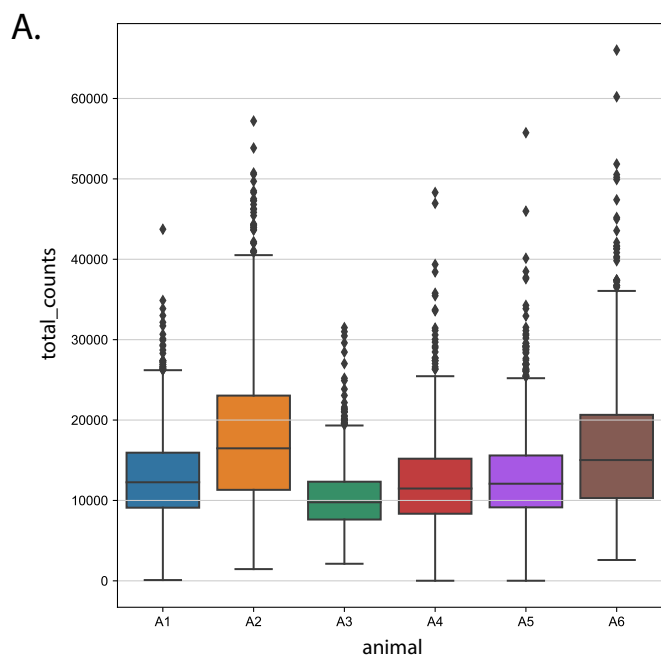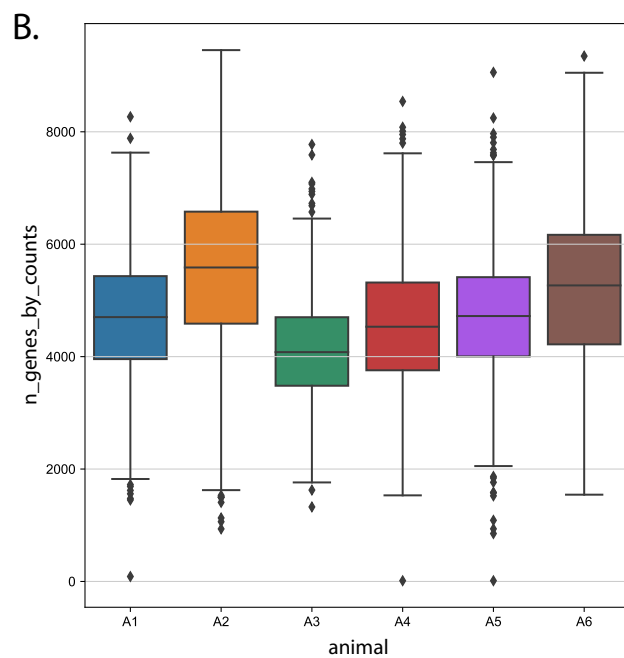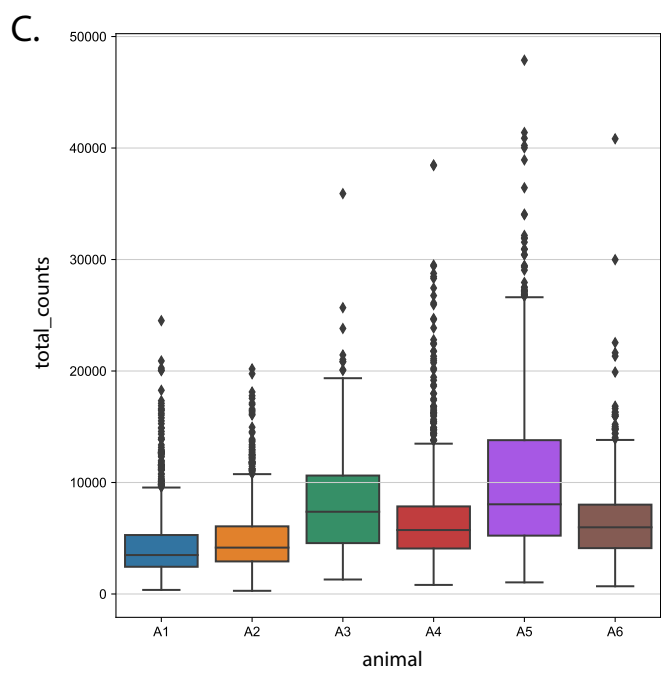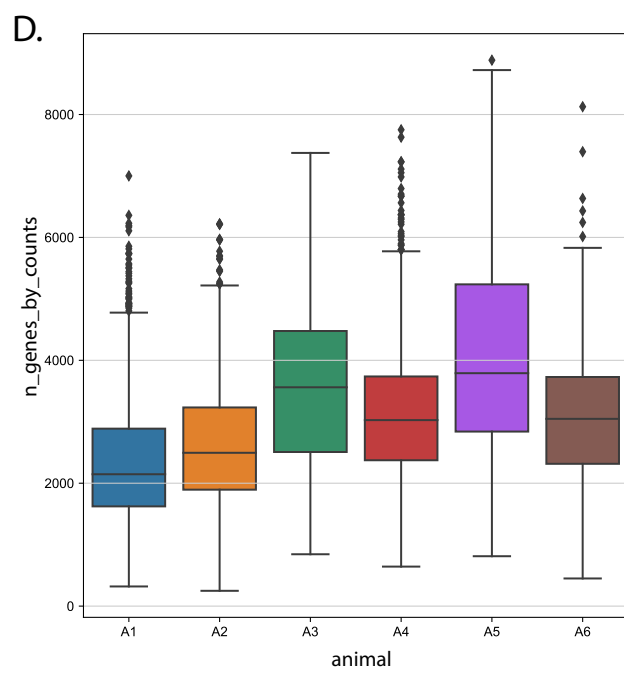

Supplement: SUPPLEMENTARY FIGURE S1 — Quality control for of the ST analysis. (A) Average number of counts per spot in each Tau22 mouse. (B) Average number of RNAs detected per spot in each Tau22 mouse. (C) Average number of counts per spot in each TauKO mouse. (D) Average number of RNAs detected per spot in each TauKO mouse. [file Data_Sheet_1.pdf]

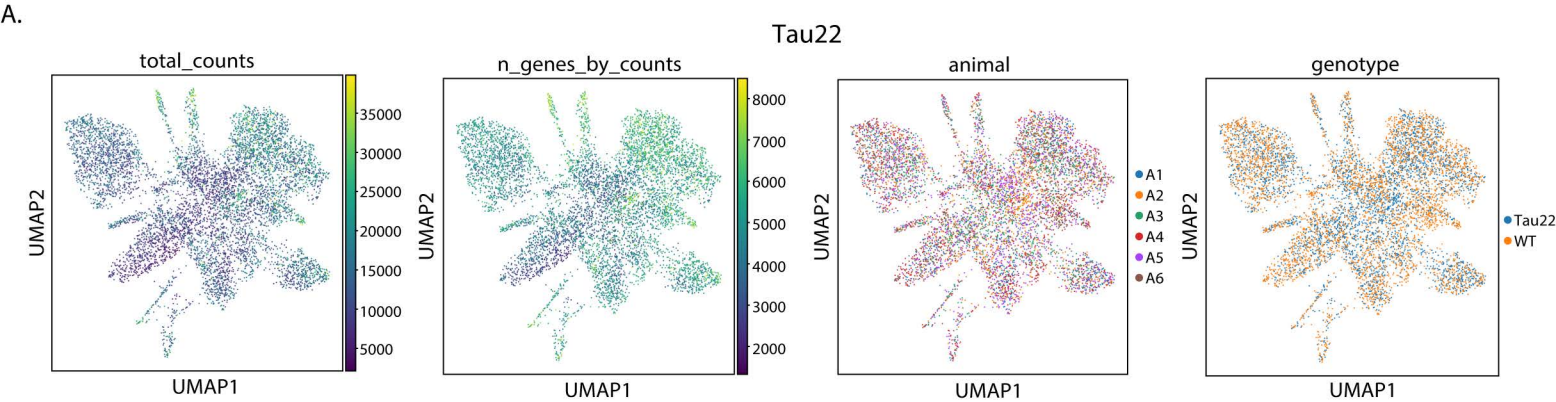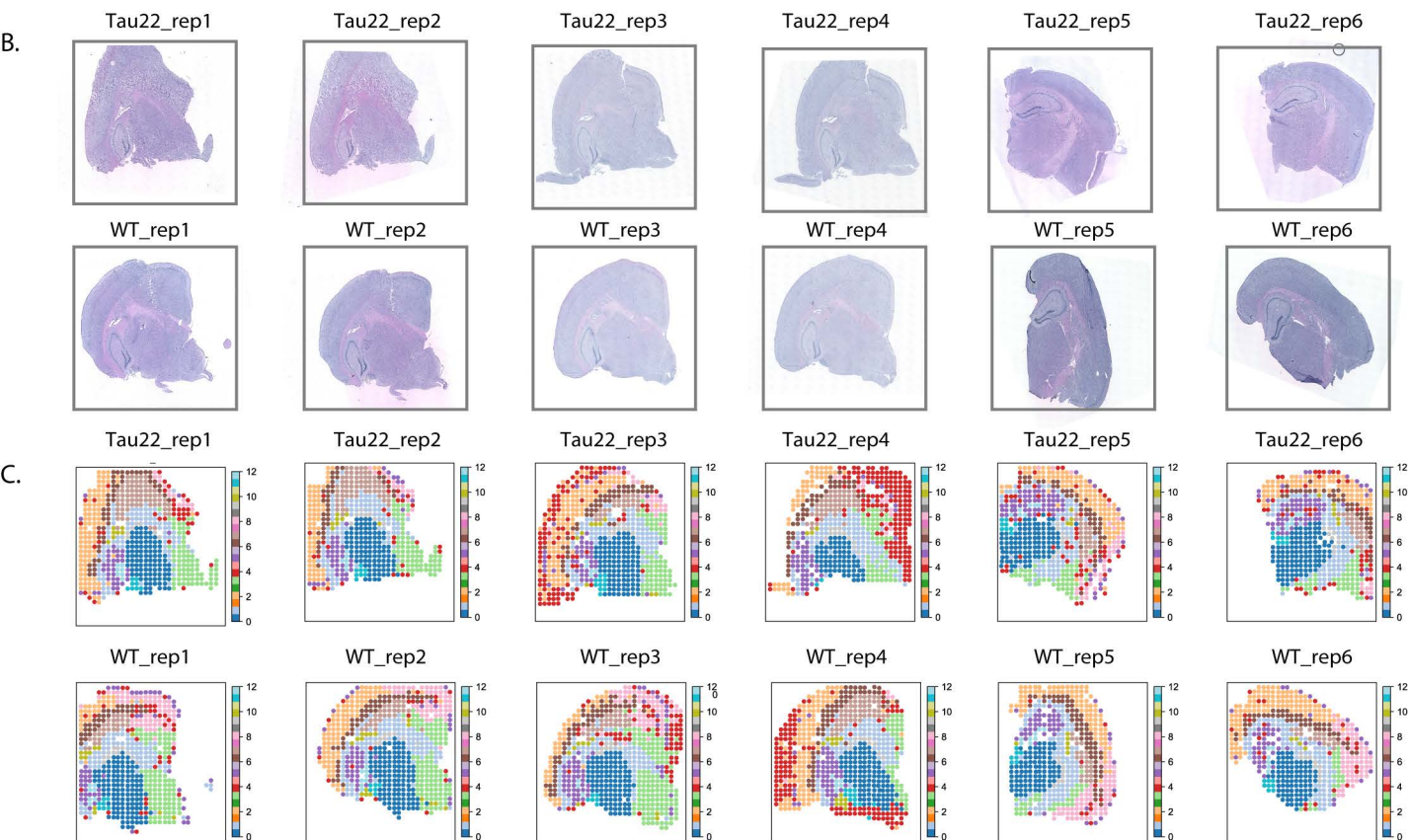

Supplement: SUPPLEMENTARY FIGURE S2 — Graphic representation of Tau22 mouse data. (A) UMAP plots colored by total number of counts per total RNAs detected, per mouse, and genotype. (B) H&E-stained brain sections for Tau22 mice. (C) Spatial representation of spots colored by cluster in Tau22 mouse brains. [file Data_Sheet_2.pdf]

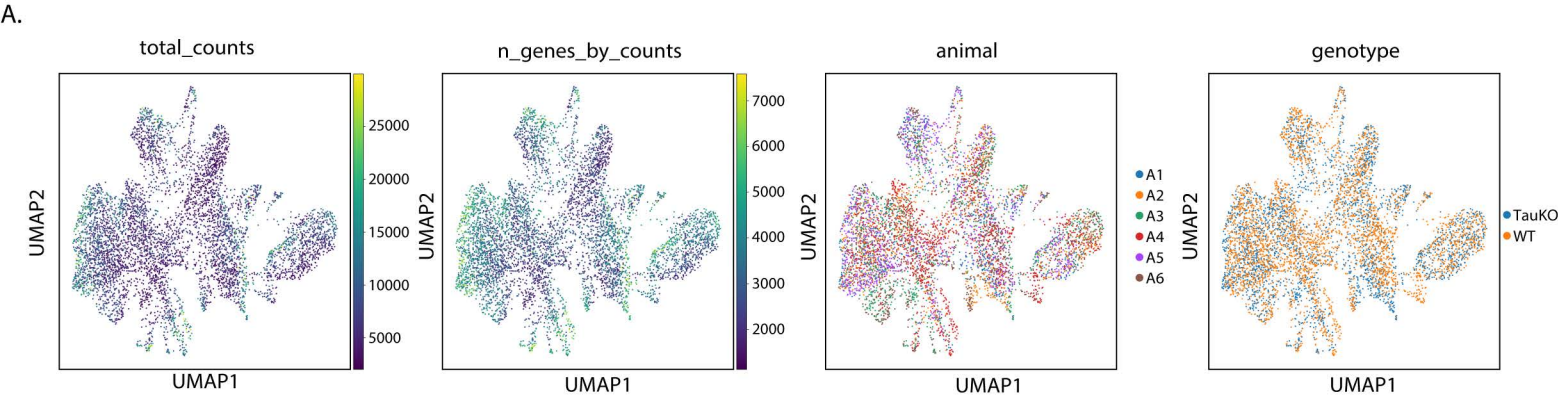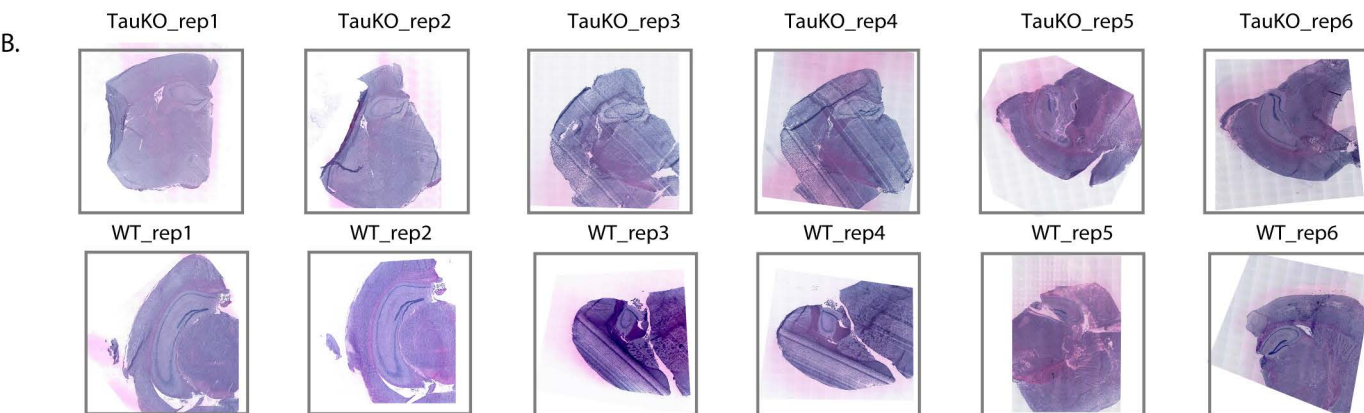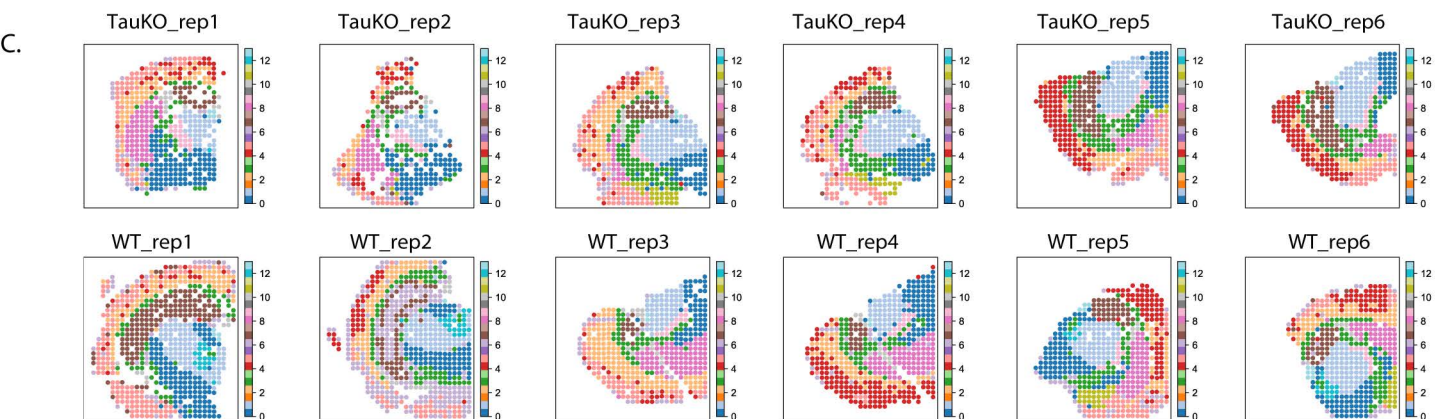

Supplement: SUPPLEMENTARY FIGURE S3 — (A) UMAP plots colored by total number of counts per total RNAs detected, per mouse and genotype. (B) H&E-stained brain sections for TauKO mice. (C) Spatial representation of spots colored by cluster in TauKO mouse brains. [file Data_Sheet_3.pdf]

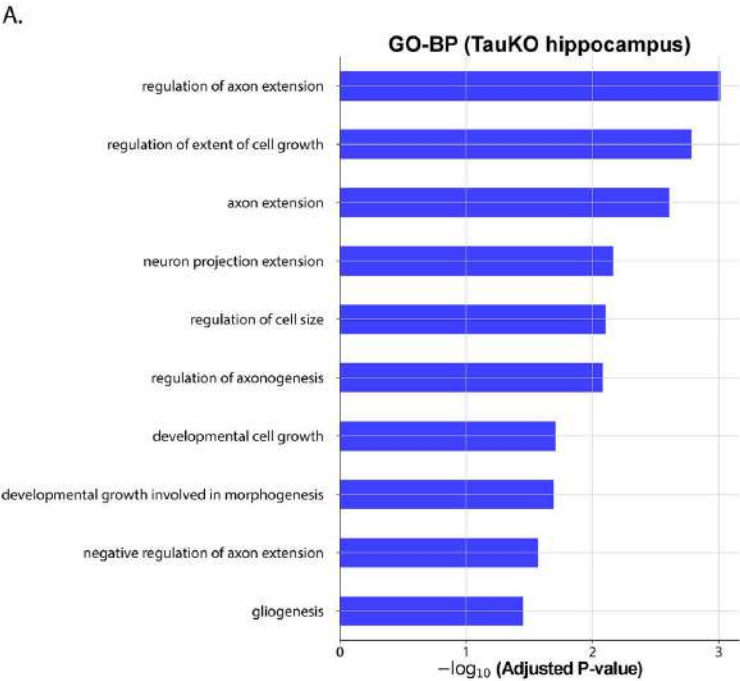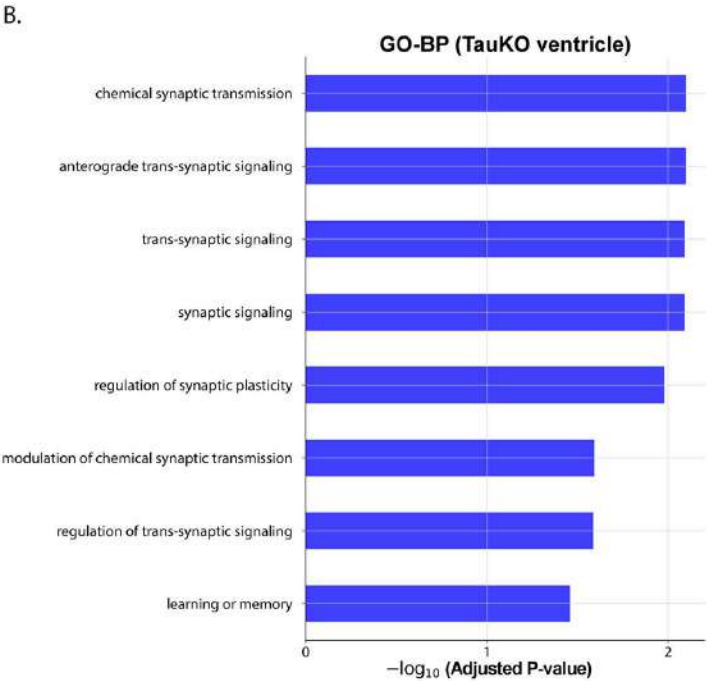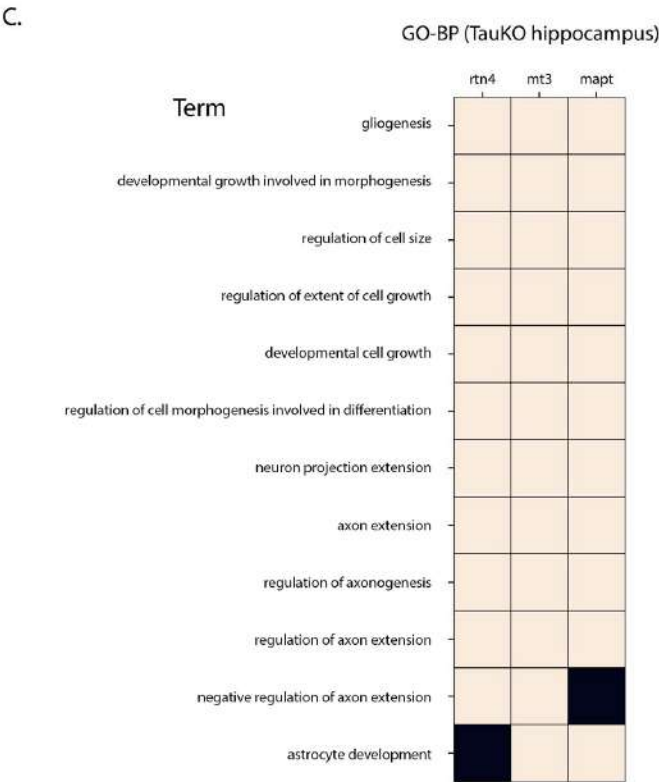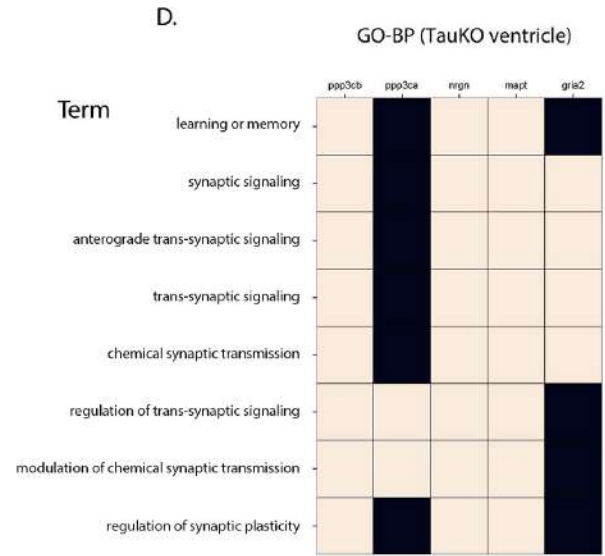

Sup Fig. 4

Supplement: SUPPLEMENTARY FIGURE S4 — RNA ontology analysis. (A) RNA set enrichment analysis (GSEA) of the top GO biological process terms from TauKO hippocampus dataset. (B) Heatmap of the significantly changed DE RNAs found in various terms. (C) GSEA of the top GO biological process terms from TauKO ventricles dataset. [file Data_Sheet_4.pdf]

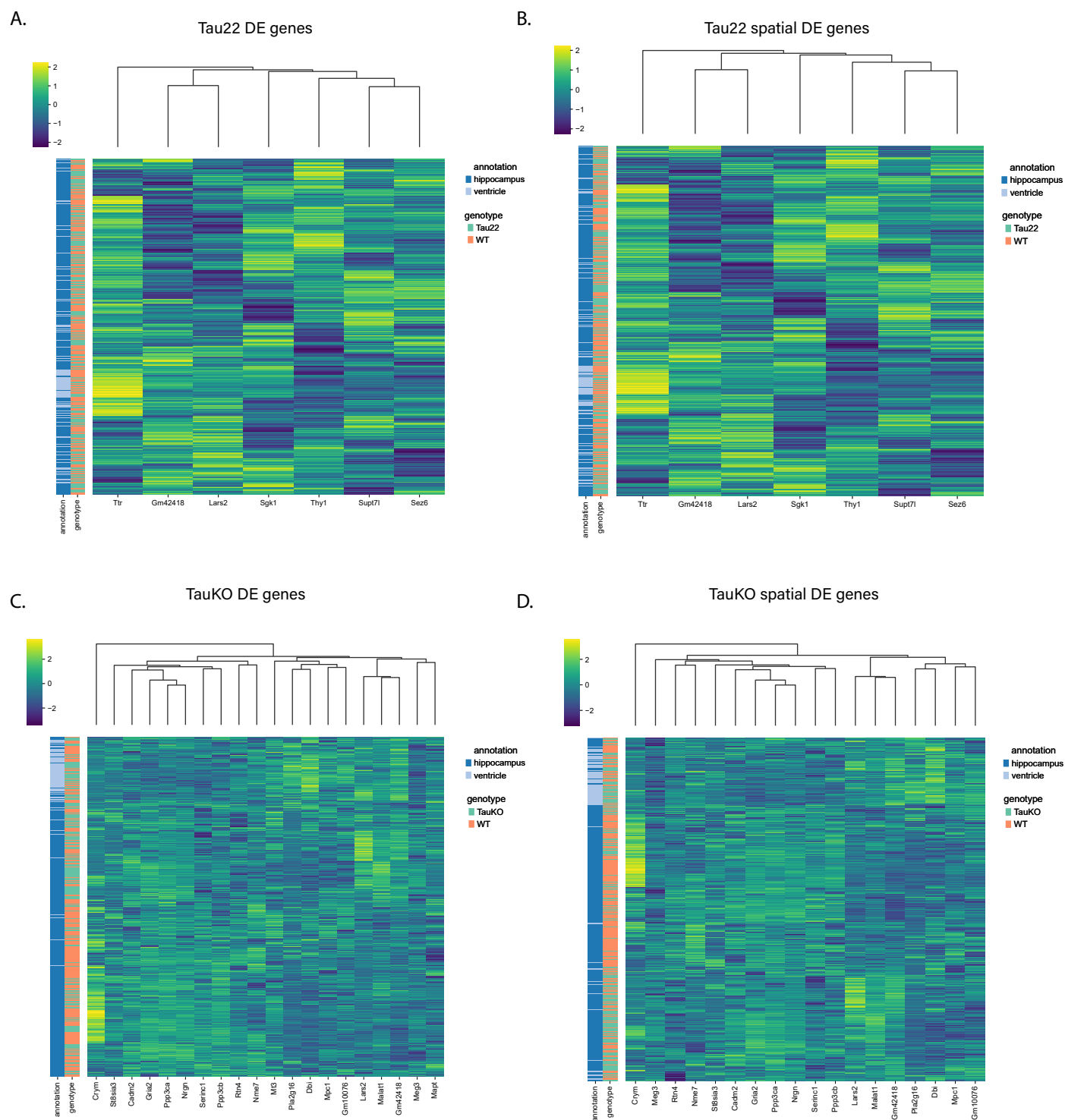

Sup Fig. 5

Supplement: SUPPLEMENTARY FIGURE S5 — Heatmaps of top Spatial DE and DE RNAs. (A) Top DE RNAs from Tau22 mice. (B) Intersection of top spatial DE and DE RNAs from Tau22 mice. (C) Top DE RNAs from TauKO. (D) Intersection of top spatial DE and DE RNAs from TauKO mice. [file Data_Sheet_5.pdf]

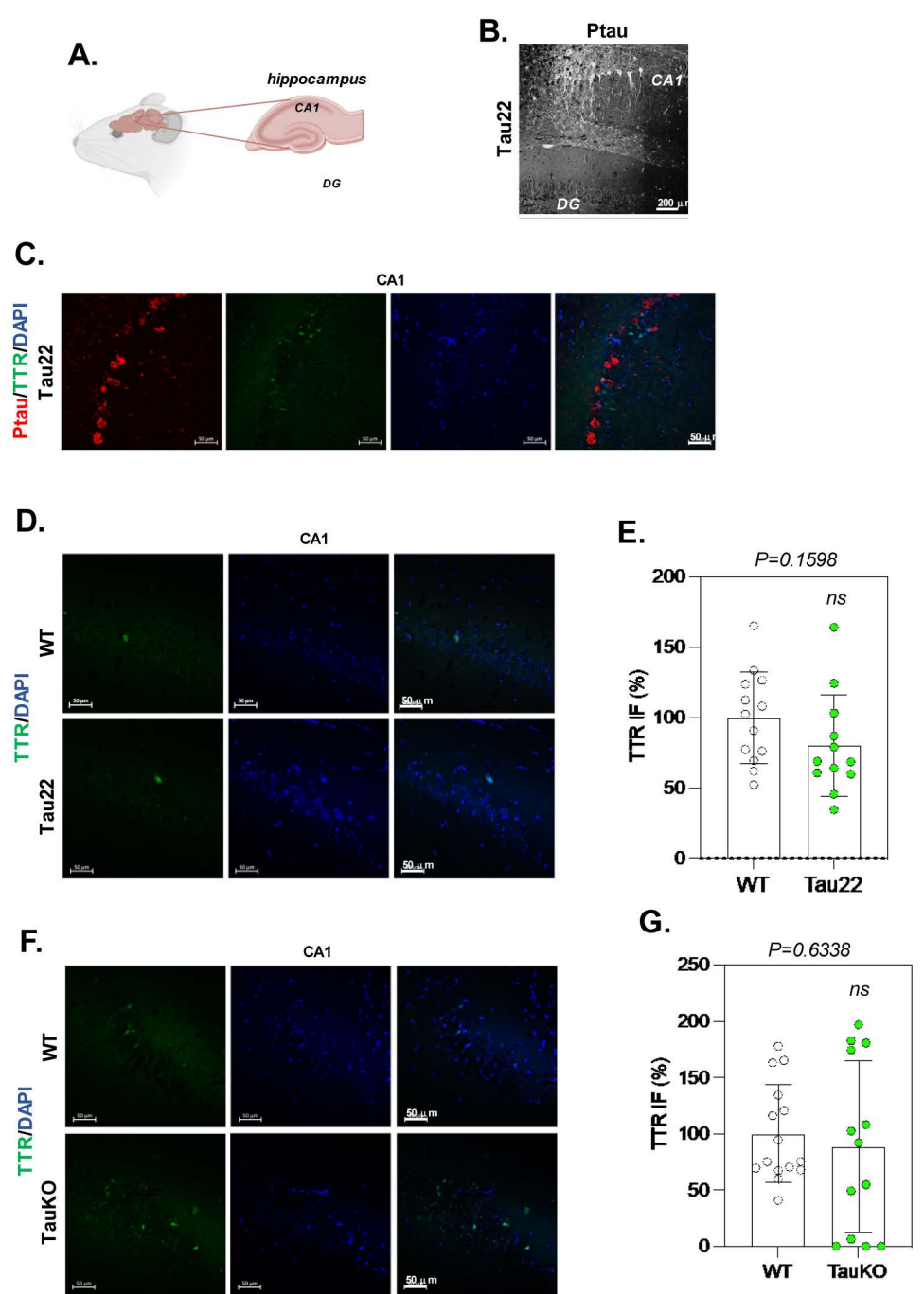

Supplement: SUPPLEMENTARY FIGURE S6 — Tau alterations do not affect TTR expression in hippocampal cells from Tau22 mouse brains. (A) Schematic representation of the hippocampus from mouse brain. (B) Representative image of sagittal sections from 12 months-old Tau22 mouse hippocampus. The section was labeled with the phospho-dependent anti-tau AT8 antibody. IF signals were analyzed by clsm (z projection). Nuclei were detected with DAPI staining. The scale bar represents 200 μm. (C) Representative images of sagittal sections from Tau22 (n = 12) mouse brains. The sections were labeled with the phospho-dependent anti-tau AT8 and the anti-TTR antibody. IF signals were analyzed by clsm (z projection). Nuclei were detected with DAPI staining. The scale bars represent 50 μm. (D) Representative images of sagittal sections from WT (n = 13) and Tau22 (n = 12) mouse brains. The sections were labeled with the anti-TTR antibody. IF signals were analyzed by clsm (z projection). Nuclei were detected with DAPI staining. The scale bars represent 50 μm. (E) The intensity of the TTR IF signals were quantified within hippocampal CA1 cells from WT (n = 13) and Tau22 (n = 12) mouse brains. Graph shows the mean of TTR fluorescence per genotype. Each biological replicate represents one mouse. Data are presented as mean ± SEM (ns: p > 0.05; Mann Whitney U test). (F) Representative images of sagittal sections from 12 months-old WT (n = 15) and TauKO (n = 13) mouse brains. The sections were labeled with the anti-TTR antibody. IF signals were analyzed by clsm (z projection). Nuclei were detected with DAPI staining. The scale bars represent 50 μm. (G) The intensity of the TTR IF signals were quantified within hippocampal CA1 cells from WT (n = 15) and TauKO (n = 13) mouse brains. Graph shows the mean of TTR fluorescence per category. Each biological replicate represents one mouse. Data are presented as mean ± SEM (ns: p > 0.05; Mann Whitney U test). [file Data_Sheet_6.pdf]

Abeta (MOAB2)/DAPI

TauKO

WT

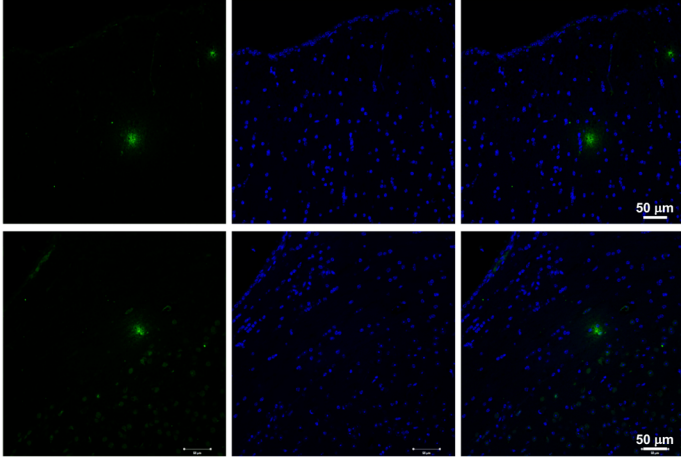

Supplement: SUPPLEMENTARY FIGURE S7 — Aβ deposits are only weakly detected in 12 months old Tau22 and WT littermate mouse brains. Representative images of sagittal sections from 12 months old WT (n = 15) and TauKO (n = 13) mouse brains. The sections were labeled with the anti-Aβ antibody MOAB2. IF signals were analyzed by clsm (z projection). Nuclei were detected with DAPI staining. The scale bars represent 50 μm. [file Data_Sheet_7.pdf]
